# P_0_-Related Protein Accelerates Human Mesenchymal Stromal Cell Migration by Modulating VLA-5 Interactions with Fibronectin

**DOI:** 10.3390/cells9051100

**Published:** 2020-04-29

**Authors:** Maria G. Roubelakis, Grigorios Tsaknakis, Feng-Juan Lyu, Ourania Trohatou, Andrew C. W. Zannettino, Suzanne M. Watt

**Affiliations:** 1 Laboratory of Biology, National and Kapodistrian University of Athens Medical School, 115 27 Athens, Greece; trohatou@bio.demokritos.gr; 2 Stem Cell Research, Nuffield Division of Clinical Laboratory Sciences, Radcliffe Department of Medicine, University of Oxford, Oxford OX3 9BQ, UK; g.tsaknakis@uoc.gr (G.T.); Lufj0@scut.edu.cn (F.-J.L.); suzanne.watt@ndcls.ox.ac.uk (S.M.W.); 3 Department of Hematology, School of Medicine, University of Crete, 70013 Heraklion, Crete, Greece; 4 South China University of Technology-the University of Western Australia Joint Centre for Regenerative Medicine Research, School of Medicine, South China University of Technology, Guangzhou 510006, China; 5 Department of Orthopedics and Traumatology, Li Ka Shing Faculty of Medicine, University of Hong Kong, 102 Pok Fu Lam Road, Hong Kong, China; 6 Adelaide Medical School, Faculty of Health and Medical Sciences, University of Adelaide, Adelaide 5005, Australia; andrew.zannettino@adelaide.edu.com; 7 Cancer Program, Precision Medicine Theme, South Australian Health and Medical Research Institute, Adelaide 5001, Australia; 8 Central Adelaide Local Health Network, Adelaide 5000, Australia

**Keywords:** human bone marrow stromal cells, PZR, SHP-2, cell adhesion molecules, fibroblasts, ITIM, migration, VLA-5, PFAK, vinculin

## Abstract

P_0_-related protein (PZR), a Noonan and LEOPARD syndrome target, is a member of the transmembrane Immunoglobulin superfamily. Its cytoplasmic tail contains two immune-receptor tyrosine-based inhibitory motifs (ITIMs), implicated in adhesion-dependent signaling and regulating cell adhesion and motility. PZR promotes cell migration on the extracellular matrix (ECM) molecule, fibronectin, by interacting with SHP-2 (Src homology-2 domain-containing protein tyrosine phosphatase-2), a molecule essential for skeletal development and often mutated in Noonan and LEOPARD syndrome patients sharing overlapping musculoskeletal abnormalities and cardiac defects. To further explore the role of PZR, we assessed the expression of PZR and its ITIM-less isoform, PZRb, in human bone marrow mesenchymal stromal cells (hBM MSC), and its ability to facilitate adhesion to and spreading and migration on various ECM molecules. Furthermore, using siRNA knockdown, confocal microscopy, and immunoprecipitation assays, we assessed PZR and PZRb interactions with β1 integrins. PZR was the predominant isoform in hBM MSC. Migrating hBM MSCs interacted most effectively with fibronectin and required the association of PZR, but not PZRb, with the integrin, VLA-5(α5β1), leading to modulation of focal adhesion kinase phosphorylation and vinculin levels. This raises the possibility that dysregulation of PZR function may modify hBM MSC migratory behavior, potentially contributing to skeletal abnormalities.

## 1. Introduction

Noonan and LEOPARD (known as Noonan syndrome with multiple lentigines) syndromes are autosomal dominant disorders that share overlapping clinical presentations, which include musculoskeletal abnormalities and malformations [1]. Over 50% of patients with Noonan syndrome and 85–90% with Noonan syndrome with multiple lentigines, respectively, carry activating or inactivating germline mutations in *PTPN11* (protein tyrosine phosphatase non-receptor 11), the gene encoding cytoplasmic Src homology-2 protein tyrosine phosphatase (SHP-2) [1]. Analysis of these mutations has hastened our understanding of SHP-2 regulatory mechanisms during homeostasis and in the context of the diseases cited above. Structurally, in its N-terminal region, SHP-2 carries two SH2 domains (N-SH2 and C-SH2) linked in tandem to a PTP (protein tyrosine phosphatase) catalytic domain [2]. Intra-molecular binding of PTP to the N-SH2 domain maintains an inhibitory switch, which places SHP-2 in a closed configuration, thereby preventing upstream interactions with tyrosine phosphorylated targets [3,4]. Mutations in the interacting regions of the N-SH2 and PTP domains, or responses to microenvironmental cues, can switch SHP-2 to an open conformation [1,2,5], where the SH2 domains have the ability to bind phosphotyrosine residues on their upstream substrates, thereby regulating cellular signaling related to cell survival, proliferation, differentiation, adhesion, spreading, or migration [6,7,8,9,10]. 

Our studies, and those of others, have demonstrated that, when tyrosine phosphorylated, P_0_-related protein (PZR) serves as a docking receptor or target for SHP-2 [11,12,13,14,15]. Human (h) PZR is a 35 kD type 1 transmembrane member of the Ig superfamily with homology to myelin P_0_ [11,13]. Intracellularly, it contains two immunoreceptor tyrosine-based inhibitory motifs (ITIMs; VIY(246)AQL and VVY(263)ADI), the phosphorylated tyrosines of which are essential for the recruitment and activation of SHP-2 [11]. Two isoforms have been detected by quantitative RT-PCR in the HS-5 hMSC cell line, *PZR* itself and an ITIM-less *PZRb*, with the former being over 20 times more abundant than the latter isoform [11]. Transfection of these isoforms individually into SHP-2 competent mouse embryonic fibroblasts (MEFs) has shown that the hPZR, but not the hPZRb, isoform accelerates the motility of these cells on fibronectin [11]. These studies also show that a mutated form of SHP-2, SHP-2(Δ46–110), in which the N-SH2 domain is deleted, fails to support human PZR mediated motility in these cells. In addition, we have cloned murine PZR and demonstrated that it modulates SHP2-mediated cell adhesion and migration on fibronectin [12]. Interestingly, while murine PZR is not expressed in pluripotent embryonic stem (ES) cells, it is upregulated in cells comprising the embryonic day E3.5 blastocyst [12]. Leading on from this, Bennett and colleagues identified PZR/Pzr as a major hyper-tyrosine phosphorylated protein and c-Src substrate in murine and zebrafish models of Noonan and/or LEOPARD syndrome. This group also demonstrated that PZR/Pzr is a target for SHP-2/Shp-2 mutants and is associated with defects in convergence and extension movements in these zebrafish during gastrulation [16,17,18,19,20]. 

In this manuscript, we demonstrate that the human *PZR* gene is expressed at a much higher level than its *PZRb* isoform in primary human bone marrow mesenchymal stromal cells (hBM MSCs). We then examined the human PZR and PZRb ability to modulate hBM MSC adhesion to, and spreading and migration on the ECM proteins, fibronectin, laminin, vitronectin, and collagens I and IV. Using siRNA knockdown technology, we found that human PZR predominately enhanced α5β1 integrin mediated migration on fibronectin in hBM MSCs. To confirm this, model systems were established with murine fibroblasts (NIH3T3 cells), which individually overexpressed either human PZR or PZRb, and where the expressed proteins could be knocked down with the appropriate siRNAs. High-resolution confocal microscopy together with immunoprecipitation and immunoblotting technologies were also used to show that human PZR interacts with the α5β1 integrin, subsequently modulating the expression of associated adhesome molecules such as phosphorylated focal adhesion kinase and vinculin. 

## 2. Materials and Methods

The materials and methods are described here in brief, and in detail in the Supplementary Materials.

### 2.1. Primary Cells

hBM MSCs were purchased from Lonza Biologics, Slough, England at passage 2 and maintained in culture in mesenchymal stem cell growth medium (MSCGM, Lonza Biologics) supplemented with 10% FCS (Gibco-BRL, Thermo Fisher Scientific, Milton Keynes, England). Cells were used up to passage 6, with the majority of experiments carried out at passage 5. In some experiments, hBM MSC were plated at a density of 14,000 cells/cm^2^ in complete MSCGM and incubated at 4, 16, and 24 h in normoxic (20% O_2_) and hypoxic (1.5% O_2_) conditions. Alternatively, cobalt chloride (CoCl_2_) (Sigma-Aldrich Ltd., St. Louis, MO, USA) was added as a hypoxia mimetic to the medium in a final concentration of 150 μM and cells incubated for 4, 16, and 24 h in normoxic (20% O_2_) conditions. 

### 2.2. Cell Lines and Stable Transfectants

The murine NIH3T3 mesenchymal and murine embryonic fibroblast (MEF) cell lines were obtained from the American Type Cell Collection (ATCC, Manassas, VA, USA) or European Collection of Cell Cultures (ECACC, Porton Down, Wiltshire, England). The murine NIH3T3 mesenchymal cell line was also used to generate human P_0_-related protein (PZR) and PZRb stable transfectants as described in Supplementary Materials and [12,21,22]. All cells were maintained in Dulbecco’s modified Eagle’s medium (DMEM; Sigma-Aldrich Ltd.) supplemented with 10% (*vol*/*vol*) FCS (Gibco-BRL). Mouse embryonic fibroblasts (MEF) stably expressing human PZR and PZRb were generated as previously described [11]. 

### 2.3. Cell Staining and Analysis 

#### 2.3.1. Cell Counts and Viability 

Cell viability was assayed by 0.4% (*wt*/*vol*) Trypan Blue exclusion or with flow cytometry using 1 μM ToPro-3 (Molecular Probes, Thermo-Fisher Scientific). 

#### 2.3.2. Antibodies and Flow Cytometric Analysis of Cell Surface Antigens 

A detailed description of the antibodies used and flow cytometry is provided in Supplementary Materials and in previous publications [11,12,21,22,23,24,25]. 

### 2.4. Hunam Bone Marrow Mesenchymal Stromal Cells (hBM MSC) Lineage Differentiation

Individual batches of hBM MSCs (passages 5–6) were analyzed for their ability to undergo adipogenic, osteoblastic, and chondrogenic differentiation in vitro as described previously [24,25]. Consistent with BM perivascular stem/stromal cells, the hBM MSCs did not express CD34, CD14 or CD45, but were strongly positive for CD90, CD146, CD73, and CD105, and exhibited osteogenic, adipogenic, and chondrogenic potential (Supplementary Figure S1).

### 2.5. Quantitative Real Time PCR (qRT-PCR)

A detailed protocol for real-time quantitative PCR analysis is provided in Supplementary Materials and in previous publications [11,12,21,22].

### 2.6. siRNA Knockdown

siRNA probes were designed for unique regions of human *PZR* and *PZRb* using the XERAGON Inc. (www.xeragon.com) web-based software (Qiagen Ltd., Manchester, England) and purchased from Dharmacon Ltd., Lafayette, CO, USA. The siRNA probe *PZR1* (5′-AATGGTACACAAGGGAAGCTC-3′) was designed to inhibit both PZR and PZRb; *PZR2* (5′-C A C C A G G G C C C AG T C A T A T A T G C-3′) and *PZR4* (5′-A A G A G T C T G C C T T C T G G A T C T-3′) were designed to inhibit PZR, whereas *PZR3* (5′-G G A T T A C A C T G G G G C C C A G T C A-3′) was designed to inhibit PZRb (Supplementary Figure S2A). Negative control siRNAs included scrambled sequences for *PZR2*, *Scr.PZR2* (5′-G G U A C U A A U G C G C G C G A C A C U A-3′) and for *PZR3*, *Scr.PZR3* (5′-G G G U C G U C C C C U G A U A C C-3′) and the control *siRNA* (5′-U A G C G A C U A A A C A C A U C A A-3′). NIH3T3, NIH3T3-hPZR, NIH-3T3-hPZRb, or hBM MSCs cells were transfected using the lipofection method (Lipofectamine 2000, Invitrogen Ltd., Thermo Fisher Scientific) described in [21,22] and Supplementary Materials. The optimization was performed by monitoring hPZR and hPZRb expression levels by flow cytometry with the WM78 monoclonal antibody and viability each day after the siRNA transfection (Supplementary Figure S2B–E). To knock down NEDD9 in the control experiments, siRNAs *NEDD9-1* and *NEDD9-2* were purchased from Dharmacon Ltd. *NEDD9-1* siRNA was a SMARTpool siRNA, a collection consisting of four individual siRNA duplexes all targeting specifically *NEDD9* (three target exon 5 and one targets exon 7), whereas NEDD9-2 was an individual siRNA duplex against exon 2 (Supplementary Figure S3A). For subsequent assays, knockdown was performed 2 and 5 days after the initial transfection for hBM MSCs and NIH3T3 cells, respectively.

### 2.7. Functional Studies 

#### 2.7.1. Coating Surfaces with Extracellular Matrix Proteins 

A detailed protocol is provided in Supplementary Materials.

#### 2.7.2. In Vitro hBM MSC Adhesion Assay

hBM MSCs, untreated, or after siRNA transfection (48 h for NEDD9 siRNAs or 72 h for PZR siRNAs) or sham transfection, were collected after accutase detachment, labeled with fluorescent dye, 2′,7′-bis-(2-carboxyethyl)-5-(and-6)-carboxyfluorescein, acetoxy methyl ester (BCECF-AM, B-1170, Molecular Probes, Thermo-Fisher Scientific) and assayed for adhesion to the relevant ECM molecule or control BSA as described in Supplementary Materials. Each experimental variable was tested in triplicate for each individual experiment and for each of three independent experiments. The percent adhesion, with or without anti-integrin antibody blocking [11], was calculated by dividing the fluorescent intensity of the test well by the average fluorescence intensity of the input control, and multiplying by 100. 

#### 2.7.3. In Vitro hBM MSC Spreading Assay 

hBM MSCs, without or following 48 h (for NEDD9 siRNAs) or 72 h (for PZR siRNAs) of Lipofectamine transfection, were seeded in serum free DMEM in 24-well plates coated with selected ECM molecules, placed in a 37 °C incubator and allowed to adhere and spread for 60 min. Phase images were taken on the Nikon Eclipse TE300 microscope (Nikon UK Ltd., London, England) using the IPLab v3.61 imaging software (Scanalytics, BD Biosciences, Oxford, England). Cells (100 per field) and a total of 10 fields were counted per group in each experiment. Cells that demonstrated a round shape with a distinct and defined edge surrounding the entire circumference of the cell were defined as “non-spreading”. Cells exhibiting visible lamellipodia/filopodia or a polygonal shape were defined as “spreading”. The percentage of spreading cells was calculated as the number spreading cells divided by the total number of cells in that field multiplied by 100.

#### 2.7.4. In Vitro hBM MSC and NIH3T3 Transfectant Migration Assays-Wound Healing Assay

NIH3T3 transfectants or hBM MSCs with or without siRNA treatment were seeded in Dulbecco′s Modified Eagle′s Medium (DMEM) with 10% FCS or complete MSCBM, respectively, in 24-well plates coated with selected ECM molecules. When confluent, a 1 mm strip of cells (or “scratch”) was removed from the center of the well with an Eppendorf pipette tip, prior to washing the monolayer and further incubation in DMEM medium with 10% FCS or in complete MSCGM. The migration of the cells into the scratch was determined by capturing images within pre-marked zones on a T-300 Nikon inverted microscope fitted with a Coolpix 900 digital camera (both from Nikon Ltd.), either immediately after the cell free scratch was made or after 6 h incubations for NIH3T3 cells and 20 h incubations for hBM MSCs at 37 °C in a 5% humidified CO_2_ incubator. The area (width of the scratch X diameter (cm^2^)) of the cell free zone was measured and used to calculate the speed of cell migration (rate of wound healing) over these time points. Two images of the migrating cells were captured per well (experimental condition) and there were triplicates for each experimental condition. Experiments were repeated independently on three occasions, following independent transfections. Since the migration assay was the longest functional assay (20 h after PZR molecules were knocked down over a 3 day period), gene silencing in hBM MSCs was shown to be maintained for this period of time using flow cytometry (Supplementary Figure S2B). For NEDD9, Western blotting was performed on transfectants at an equivalent time point (i.e., 3 days after transfection) to confirm that silencing was maintained during this time period of transfection plus assay. Supplementary Figure S3B shows a representative Western blot which indicates a reduction in NEDD9 expression after siRNA knockdown prior to the performance of the adhesion and migration assays (Supplementary Materials and Methods). The effects on subsequent cell migration is shown in Supplementary Figure S3C).

### 2.8. Immunoprecipitation and Immunoblotting

Cell biotinylation is described in detail in Supplementary Materials. In brief, cell surface biotinylation was performed as appropriate using the FluoReporter cell surface biotinylation kit (Invitrogen Ltd., Thermo Fisher Scientific). 

#### 2.8.1. Cell Lysis and Immunoprecipitation 

hBM MSCs, and NIH3T3 or MEF cell lines without or expressing human PZR or PZRb were cultured on fibronectin, washed in PBS, and then lysed in RIPA lysis buffer (all reagents from Sigma-Aldrich Ltd.) as described in Supplementary Materials and Methods. Immunoprecipitation was carried out with WM78 mAb or isotype control mAbs (mIgG1; DakoCytomation Ltd., Glostrup, Denmark) or rabbit anti-human PZR or IgG (Cell Signaling, Leiden, The Netherlands) and protein G Sepharose and analyzed for protein content using the Bio-Rad Dc Protein Assay kit (see Supplementary Materials). All other cell lysis (e.g., for NEDD9 and its alpha-tubulin control Western blots) was carried out using treatment buffer (75mM Tris-HCL, pH6.8, 3.8% (*wt/vol*) SDS, 4M Urea (*wt/vol*), β-mercaptoethanol (BME), and 20% (*vol/vol*) glycerol (all from Sigma-Aldrich Ltd.).

#### 2.8.2. Gel Electrophoresis and Western Blotting

The samples (10–50 μg of protein) were loaded onto 10%, 12%, or 4–12% NuPAGE Bis-Tris gels and then subjected to SDS-PAGE, before transfer to nitrocellulose or PVDF membranes (Gibco-BRL, Thermo Fisher Scientific), the membrane blocked with milk powder and then as appropriate incubated with primary antibody followed by HRP goat anti-rabbit or anti-mouse IgG or streptavidin (all in 1/2000 dilution). The protein signal was detected using the Supersignal West Dura Extended Duration Substrate (Pierce Biotechnology Inc., Thermo Fisher Scientific) enhanced chemiluminescent (ECL) kit to detect for the HRP-conjugated secondary antibody as detailed in [11] and Supplementary Materials. Normalization was performed using antibody against alpha-tubulin (Sigma-Aldrich Ltd.) or beta-actin (BD Biosciences). Alternatively, the nitrocellulose membrane was blocked with LICOR blocking buffer (Li-COR, Lincoln, NE, USA) and probed with rabbit anti-human PZR (1:500) overnight at 4 °C. Next, the secondary IRDye 800CW goat anti-rabbit IgG (1:5000; Li-COR), was incubated with the membrane for 1.5 h, and the membrane imaged. The membrane was reprobed with an anti-human beta-actin antibody, prior the addition of secondary IRDye 680RD donkey or goat anti-mouse IgG (Li-COR) for 1 h at room temperature. After washing, detection of positive signal was achieved using the Odyssey CLx Infrared Fluorescent imager plus (Li-COR). Normalization was performed using antibody against beta-actin (BD Biosciences). Densitometry analysis was performed using Li-COR Image studio, and the PZR/PZRb protein densities normalized to beta-actin.

### 2.9. Confocal Microscopy

#### 2.9.1. Co-Localization Studies for Integrins and PZR Isoforms

First, 1–5 × 10^5^ hBM MSCs or NIH3T3 transfectants/well were cultured overnight in four chamber polystyrene vessel tissue culture treated glass slides (BD Biosciences) coated with 20 μg/mL fibronectin overnight at 4 °C. Slides were incubated with primary antibodies or isotype specific controls followed by appropriately conjugated streptavidin, goat anti-mouse (m) IgG1, or goat anti-rabbit secondary antibodies (Molecular Probes, Thermo-Fisher Scientific) and cells then fixed with 3% paraformaldehyde. Images were acquired using an automated Zeiss 510 confocal microscope (Carl Zeiss Microimaging Inc., Thornwood, NY, USA) fitted with HeNe543, HeNe 633 and argon 488 lasers. The fluorescent images were exported and further processed using Imaris 3.3 software (Bitplane AG, Zürich, Switzerland). The percentage of co-localization of the proteins of interest was determined for antibody stained cells at the interface with the scratch in the wound healing migration assay using the Imaris co-localization module of Imaris software (see Supplementary Materials for full details). 

#### 2.9.2. Staining for Phosphorylated-Focal Adhesion Kinase (PFAK) and Vinculin

First, 2 × 10^4^ hBM MSCs/well were cultured on glass coverslips in a 24-well plate, precoated with or without 20 μg/mL fibronectin. For each siRNA probe, 20 μM siRNA was diluted in 50 μL OptiMEM medium, whilst 2 μL Lipofectamine 2000 was diluted in 50 μL OptiMEM. The tubes were incubated separately for 15 min at room temperature. The siRNA and Lipofectamine 2000 were combined and incubated a further 15 min. The siRNA/Lipofectamine mix was then gently added to each well with total volume of 500 μL. After 6 h, the media were changed to 500 μL 20% DMEM. After 48–72 h, siRNA knockdown cells were fixed with 4% (*w/v*) paraformaldehyde in PBS pH 7.4, for 20 min at room temperature and then were permeabilized and blocked with 0.1% (*v/v*) Triton × 100 solution and 5% serum (Sigma-Aldrich Ltd.) in PBS for 30 min at room temperature. Transfected hBM MSCs were then incubated with rabbit anti-human PFAK (FAK-pY^397^) or mouse anti-human vinculin antibodies overnight at 4 °C and finally with the secondary goat anti-rabbit Alexa-546 or goat anti-mouse Alexa-488, respectively (Molecular Probes, Thermo-Fisher Scientific), for 1 h at room temperature. DAPI staining was used for visualization of nuclei before mounting them with Dako Fluorescent Mounting Medium. Fluorescent specimens were visualized and photographed with a confocal laser-scanning microscope (TCS SP5 Confocal System, Leica Biosystems, Nussloch, Germany). Images were processed and quantified with Image J version 1.43 software.

## 3. Statistics

Unless otherwise stated, data are presented as means ± S.E.M. from multiple independent experiments (*n* ≥ 3). Statistical significance was tested, as appropriate and as specified in the results, figures, or figure legends, using Student’s *t*-test for comparison of two conditions and one way ANOVA with the Tukey’s HSD post hoc test for comparison of ≥3 conditions and GraphpadPrism 8 software. Statistical differences of *p* < 0.05 were considered significant.

## 4. Results

### 4.1. P_0_-Related Protein (PZR) is the Predominant Isoform on hBM MSCs

**Figure 1:**
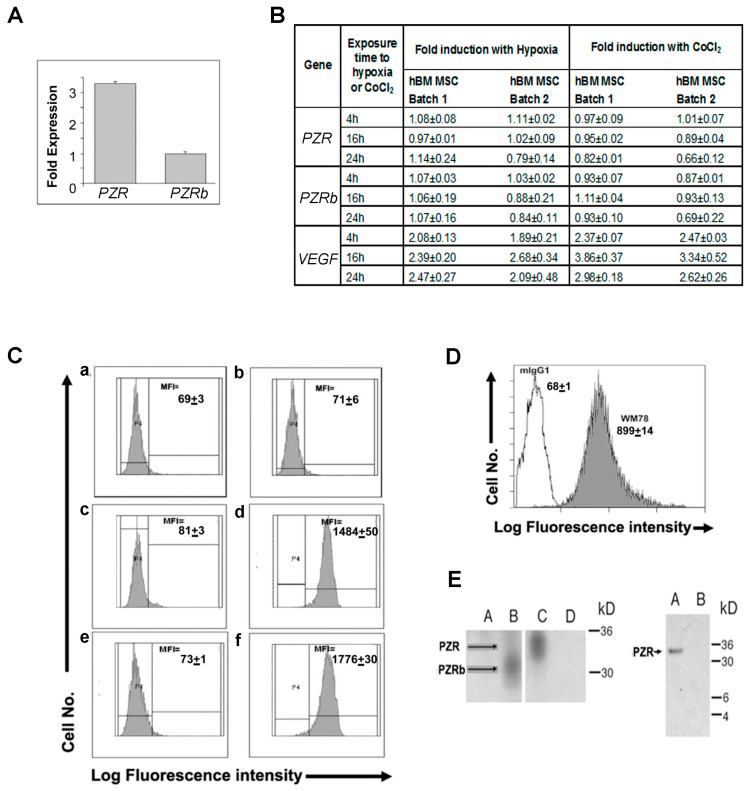
Differential human P_0_-related protein (PZR) and PZRb expression on hBM MSCs. (**A**) Q-RT-PCR of *PZR* and *PZRb* using 3 different hBM MSC batches (mean ± S.E.M.) and human *PZR* and *PZRb* primers and probes described in Section 2.5. (**B**) Q-RT-PCR of *PZR*, *PZRb*, and the positive control *VEGF* transcripts in two independent batches of hBM MSC in normoxia (20% O_2_) with or without 150 μM CoCl_2_ or hypoxia (1.5% O_2_) for 4, 16, and 24 h. Values are means ± S.E.M. of triplicate assays. (**C**) Flow cytometric histograms of human PZR and PZRb protein expression after mIgG1 (panels **a**, **c**, and **e**) or WM78 (panels **b**, **d**, and **f**) staining of MEF cells either untransfected (**a**,**b**) or expressing hPZR (**c**,**d**) or hPZRb (**e**,**f**) plus Alexa-488 goat anti-mouse IgG1 antibody. P2 is the gate set against the isotype control. The median fluorescence intensity (MFI) of cells in the positive gate is shown on each histogram. Values above are means ± S.E.M. for triplicate assays. (**D**) Representative FACS histogram of hBM MSCs staining with WM78 compared to the mIgG1 negative control stained as above. MFIs are means ± S.E.M. of three independent experiments using three different batches of hBM MSCs. (**E**) Immunoprecipitation of human PZR isoforms using WM78. Left. Biotinylated MEF cells expressing human PZRb (lanes **A** and **B**) and human PZR (lanes **C** and **D**) were immunoprecipitated with the mIgG1 (lanes **A** and **D**) or WM78 (lanes **B** and **C**) and then Western blotted using streptavidin-HRP. Right. Immunoprecipitation of human PZR isoforms from surface biotinylated hBM MSCs (lane **A**) with WM78 or mIgG1 prior to electrophoresis and Western blotting using HRP-streptavidin.

Since *PZR* is more highly expressed at the mRNA level than its *PZRb* isoform in the hBM MSC line, HS-5 [11], we analyzed the expression of these isoforms in hBM MSCs by RT-PCR. *PZR* was the predominant transcript, being over three-fold (3.2 ± 0.1; mean ± S.E.M.; *n* = 3 donors) more abundant than *PZRb* (Figure 1A). The average Ct values for *PZR* and *PZRb* in these cells were 26.40 ± 0.29 and 28.33 ± 0.24, respectively. Although the ΒΜ contains hypoxic niches [26] and hypoxia enhances hBM MSC proliferation [25], no significant changes in *PZR* or *PZRb* gene expression were observed when hBM-MSCs were subjected to hypoxia (1.5% O_2_; e.g., 24 h *PZR p* = 0.53, *PZRb p* = 0.52) or the hypoxia mimetic CoCl_2_ under normoxic conditions (20% O_2_; e.g., 24 h *PZR p* = 0.12, *PZRb p* = 0.11) for 4, 16, and 24 h compared to normoxia alone (20% O_2_; e.g., 24 h *VEGF* hypoxia *p* < 0.005, *VEGF* CoCl_2_ *p* < 0.05 and using VEGF as an hypoxia-responsive control (Figure 1B; Student’s *t*-test). For protein detection, MEF cells stably expressing human PZR or PZRb [11] served as controls. Both the control MEF-PZR and -PZRb transfectants and the hBM MSCs stain with the WM78 mAb, which identifies the common extracellular domain of the human PZR isoforms (Figure 1C,D) [11]. Immunoprecipitation and Western blotting of PZR or PZRb, respectively, from the MEF-hPZR and -hPZRb transfectants revealed bands of ≈35 kD for PZR and ≈30 kD for PZRb (Figure 1E left). Immunoprecipitation and Western blotting of hBM MSC lysates with the WM78 mAb revealed that PZR was the predominant protein isoform expressed in hBM MSCs (Figure 1E right).

### 4.2. Differential Interaction of hBM MSCs with ECM Substrates

**Figure 2:**
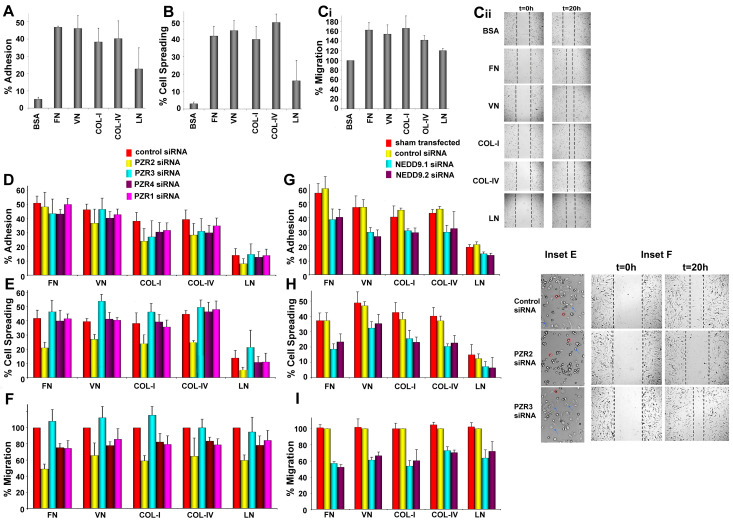
Effects of PZR on hBM MSCs in regulating adhesion to and spreading and migration on ECM molecules. (**A**) BCECF-labeled hBM MSC adhesion to BSA, FN, VN, COL-I, COL-IV, and LN after 30 min incubation. (**B**) hBM MSC spreading on FN, VN, COL-I, COL-IV, LN, or BSA for 60 min, then phase contrast images taken and percentages of spreading cells calculated. Cells adhering or spreading were calculated as the mean percentage + S.E.M. of input cells. (**Ci**) hBM MSC migrating on FN, VN, COL-I, COL-IV, LN, or BSA for 20 h. Left: Cell migration on BSA was normalized to 100% and percentage increase in migration on ECM substrates compared to this normalized value. (**Cii**) Representative phase contrast images of hBM MSC migration at 0 (left panel) and 20 h (right panel) after initiating the migration assay. Black dashed lines mark the migratory area. *t* = hours after initiation of migration. (**D**–**F**) Respective adhesion, spreading, and migration assays were repeated after hBM MSCs were treated with *PZR2-, PZR3-, PZR4*-, and *PZR1-* or control siRNAs (see Section 2.6). In (**F**), hBM MSCs treated with *PZR* and/or *PZRb* siRNAs were compared to control siRNA transfected cells, which were normalized to 100%. Representative phase contrast images of hBM MSC spreading (Inset **E**) and migration at 0 (left panel) and 20 h (right panel) after initiating the migration assay (Inset **F**) following knockdown with control, *PZR2,* and *PZR3* siRNAs. Black dashed lines mark the migratory area. *t* = hours after initiation of migration. (**G**–**I**) Respective adhesion, spreading, and migration assays were repeated after hBM MSCs were treated with *NEDD9.1*- or *NEDD9.2*- or control siRNAs (see Supplementary Materials and Methods). In (**G**), hBM MSCs treated with *NEDD9.1*- or *NEDD9.2*-siRNAs were compared to control siRNA transfected cells, which were normalized to 100%. Sham transfected cells lacking specific siRNAs were also tested. Values are means ± S.E.M. (*n* = 3 independent experiments using three different batches of hBM MSCs).

Our previous studies have shown that human PZR, but not PZRb, facilitates migration of SHP-2 competent murine MEF transfectants on fibronectin [11]. To further define PZR function on hBM MSCs, we first investigated the adhesive, spreading, and migratory properties of hBM MSCs to/on ECM proteins, fibronectin (FN), vitronectin (VN), collagen I (COL-I), collagen IV (COL-IV), and laminin (LN). hBM MSCs adhered well to four of the five ECM proteins tested within 30 min [Figure 2A: FN (46.89% ± 0.55%), VN (46.18% ± 7.38%), COL-I (38.39% ± 7.96%), COL-IV (40.24% ± 10.25%, *p* < 0.0005, one way ANOVA)] and to a lesser extent, but non-significantly, to laminin (22.77% ± 12.04%, *p* = 0.1367, Tukey’s HSD post hoc test) when compared with the non-ECM control BSA. As shown in Figure 2B, hBM MSCs showed a significant increase in their spreading ability on all ECM substrates tested, except LN, when compared to BSA (FN: *p* < 0.001, VN: *p* < 0.0005, COL-I: *p* < 0.001, COL-IV: *p* < 0.0005, LN: *p* = 0.2904; Tukey’s HSD post hoc test) within 60 min of plating the cells. Cell migration was measured as shown in Figure 2C. hBM MSCs migrated more rapidly on all five ECM proteins compared to BSA (FN: 1.62 ± 0.18-fold; VN: 1.54 ± 0.21-fold; COL-I: 1.66 ± 0.23-fold; COL-IV: 1.42 ± 0.09-fold; LN: 1.2 ± 0.04-fold; *p* < 0.005, one way ANOVA). These results demonstrate that of the five ECM proteins tested, hBM MSCs showed the highest adhesion to and spreading and migration on fibronectin, vitronectin, and collagens I and IV, when compared to laminin.

### 4.3. Knockdown of Human PZR Principally Reduces hBM MSC Migration on ECM Substrates

In order to evaluate the function of human PZR in this adhesion, spreading and migration, hBM MSCs were transfected with two different *PZR* siRNAs, *PZR2* and *PZR4*, and one *PZR/PZRb* siRNA, *PZR1*, and the functional effects of *PZR* knockdown compared with *PZRb* siRNA (*PZR3*) and control siRNA transfected cells (Supplementary Figures S2 and S4; Figure 2D–F,Inset 2E,2F). As described in the Supplementary Materials, neural precursor cell expressed developmentally downregulated protein 9 (*NEDD9*) siRNAs were used as a positive control for these studies as we have previously demonstrated that knocking down NEDD9 reduced adhesion to, and spreading and migration on, the ECM substrates tested [22] (Supplementary Figures S3 and S5; Figure 2G–I). 

Notably, for the adhesion assay, knocking down the human PZR isoforms did not affect cell proliferation or viability. However, compared to control siRNA, there was a substantial decrease in cell surface staining with the WM78 Mab after transfection of hBM MSCs with *PZR1* and the two *PZR* specific siRNAs, *PZR2* and *PZR4* (by ≈70%, ≈65%, and ≈65%; *p* < 0.0001 for all when the sham transfected or siRNA controls were compared to *PZR1, 2,* and *4* siRNAs using one way ANOVA, with Tukey’s HSD post hoc test for multiple comparisons), and no significant reduction (≈9%; *p* = 0.4414 for both controls versus *PZR3* siRNA) when hBM MSCs were treated with the *PZRb* specific siRNA, *PZR3* (Supplementary Figure S2). Such a minor reduction in the expression levels of the PZRb isoform after treatment of cells with *PZR3* siRNA can be attributed to the low expression of PZRb in hBM MSCs. There was no statistical difference in the adhesion of sham-transfected hBM MSCs to fibronectin, vitronectin, collagens I and IV, and laminin compared to the siRNA control using one way ANOVA as above (*p* = 0.5748, *p* = 0.3571, *p* = 0.3044, *p* = 0.3596, *p* = 0.4985) (Figure 2D), although a significant reduction in adhesion was generally observed with *NEDD9* siRNAs on each ECM substrate (FN: *p* < 0.05, VN: *p* < 0.05, COL-I: *p* < 0.005, COL-IV: *p* < 0.05, LN: *p* < 0.0005) (Figure 2G; Supplementary Figure S3).

The ability of *PZR*-specific siRNAs to modulate hBM MSC spreading on different ECM components was next tested, by plating cells transfected with *PZR1, PZR2, PZR3,* and *PZR4* siRNAs or cells treated with control siRNA on the ECM substrates for 60 min. One way ANOVA with Tukey’s HSD post hoc test was used for statistical analyses. Treatment of cells with *PZR2* and *PZR4* siRNAs decreased expression of PZR in hBM MSCs by over 50% (respectively, *p* < 0.001 and *p* < 0.005 compared to the sham transfected cells; and *p* < 0.0001 and *p* < 0.001 compared to the control siRNA), while *PZR1* siRNA decreased PZR expression levels by ≈66% (*p* < 0.01 and *p* < 0.005 compared to sham transfectants and control siRNA, respectively). In contrast, *PZR3* treatment led to a non-significant decrease of ≈12% (*p* = 0.3319 and *p* = 0.6559 for the respective controls). Notably, knockdown of the *PZR* isoforms did not affect cell numbers or viability of the cells (Supplementary Figure S4A–C). While there was a trend for reduced spreading after PZR knockdown (Figure 2E,Inset 2E), this only reached statistical significance upon *PZR2* siRNA treatment compared to control siRNA treatment and occurred for FN, VN, and COL-IV (FN: *p* < 0.05, VN: *p* < 0.05, COL-I: *p* = 0.0932, COL-IV: *p* < 0.005, LN: *p* = 0.4790, Tukey’s HSD post hoc test; Figure 2E). In contrast, both NEDD9 siRNA treatments significantly and consistently decreased hBM MSC spreading on the FN, VN, COL-I, and COL-IV tested (Figure 2H; Supplementary Figure S5). 

The migration assay was also performed with or without human *PZR* siRNA or human *NEDD9* knockdown (Supplementary Figures S2, S3 and S4D–F). Introduction of *PZR1, PZR2,* and *PZR4* siRNAs into the hBM MSCs significantly reduced cell surface expression of PZR or PZRb (*PZR1*: ≈68%, *p* < 0.0001 for both; *PZR2*: ≈59%, *p* < 0.0001 for both; *PZR3*: ≈8%, *p* = 0.1247 and *p* = 0.3479; *PZR4*: ≈65%, *p* < 0.0001 for both, in comparison to the sham transfectants and siRNA controls, respectively, using one way ANOVA with Tukey’s HSD post hoc test for statistical analyses; Supplementary Figure S4D–F). The *PZR1, PZR2,* and *PZR4* knockdown inhibited hBM MSC migration on fibronectin by ≈26% (*p* < 0.05), 50% (*p* < 0.0001), and ≈25% (*p* < 0.05; all Tukey’s HSD post hoc test), respectively, compared to the control siRNA (Figure 2F,Inset 2F). No significant difference was observed between *PZR3* transfected cells compared to control siRNA (*p* = 0.7640, Tukey’s HSD post hoc test). When other ECM proteins were tested, knockdown of *PZR* also reduced hBM MSC migration on these substrates, but this was generally less effective than on fibronectin. When cells were allowed to migrate on vitronectin-coated plates, *PZR2* siRNA silencing significantly reduced migration by 35% (*p* < 0.01), and *PZR1* and *PZR4* siRNAs non-significantly by 15% (*p* = 0.3681) and 23% (*p* = 0.0873; all Tukey’s HSD post hoc test), respectively (Figure 2F). On collagen I, *PZR2* siRNA treatment significantly inhibited hBM MSC migration by 41% (*p* < 0.05) compared to control siRNA transfected cells, while *PZR4* and *PZR1* reduced migration non-significantly by 18% (*p* = 0.3756) and 21%, respectively (*p* = 0.2606; all Tukey’s HSD post hoc test). On collagen IV, *PZR2* siRNA treatment reduced the mean hBM MSC migration to similar levels as observed on vitronectin (36%; *p* < 0.05), while *PZR1* and *PZR4* siRNAs had a milder, but non-significant, effect on hBM MSC migration by 22% (*p* = 0.2390) and 17% (*p* = 0.4379; all Tukey’s HSD post hoc test), respectively. On laminin, *PZR2* siRNA had a similar effect as on collagen I reducing migration up to 40% (*p* < 0.05), whereas *PZR1* and *PZR4* siRNAs inhibited migration, non-significantly, by 16% (*p* = 0.4832) and 22% (*p* = 0.2116; all Tukey’s HSD post hoc test), respectively. No inhibition of migration was observed after *PZR3* siRNA transfection, with *p* values ranging from 0.5047 to 1.0119. Notably, *NEDD9* siRNAs significantly inhibited adhesion and migration of hBM MSCs on all substrates and spreading on FN, VN, COL-I, and COL-VI. (Figure 2G–I). In summary, these data indicate that PZR on hBM MSCs most consistently regulates the rate of their migration on fibronectin. 

### 4.4. NIH3T3 Stable Transfectants Expressing Human PZR Show Enhanced Migration on Fibronectin

**Figure 3:**
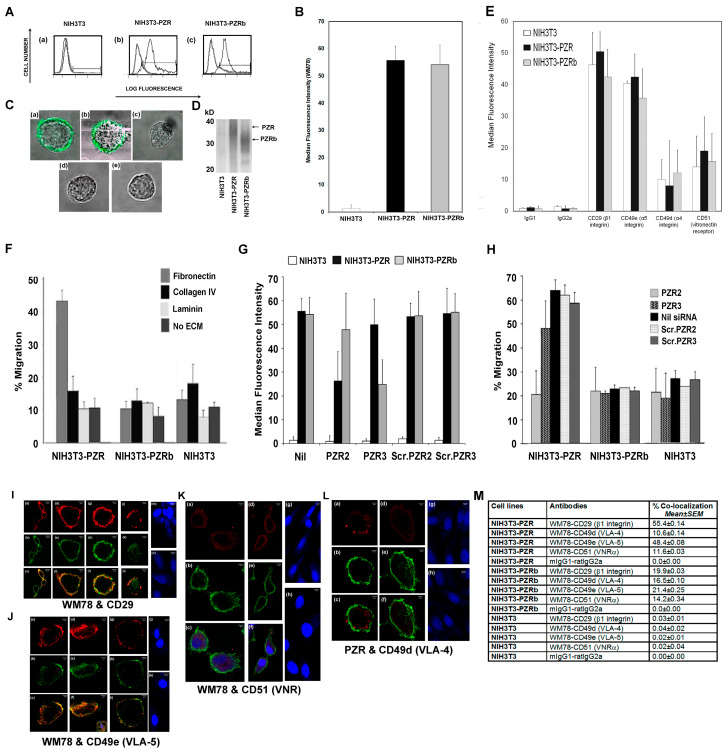
Human PZR regulates the migration of murine NIH3T3-PZR transfectants on fibronectin. (**A**) Flow cytometric histograms showing the level of expression of human PZR and PZRb as analyzed using WM78 plus FITC-goat anti-mIgG1 staining of NIH3T3 (**a**), NIH3T3-PZR (**b**), and NIH3T3-PZRb (**c**) (black outlined histograms). Grey outlined histograms are isotype matched mIgG1 negative control staining. (**B**) Graphical representation of WM78 staining in (**A**) showing MFI values as means ± S.E.M. for triplicate stains. (**C**) Representative WM78 followed by FITC-goat anti-mIgG1 staining (green) of NIH3T3-PZR stable transfectants (**a**), NIH3T3–PZRb stable transfectants (**b**), and mock transfected NIH3T3 cells (**c**) and analysis using confocal microscopy. Panels (**d**) and (**e**) show a lack of surface staining of NIH3T3-PZR and NIH3T3-PZRb cells with FITC-goat anti-mIgG1 FITC as negative controls. (**D**) Western blots of human PZR isoforms from NIH3T3 transfectants. Biotinylated cell lysates were immunoprecipitated with WM78, separated by 12% polyacrylamide gel electrophoresis and proteins detected with streptavidin alkaline phosphatase (SA-HRP). (**E**) NIH3T3 and NIH3T3 stably expressing human PZR or PZRb were stained with the biotin-conjugated rat anti-mouse -CD29, -CD49e, -CD49d, -CD51, or rat isotype controls, followed by Alexa 546-streptavidin and analyzed by flow cytometry. For (**B**) and (**E**), values represent means ± S.E.M. MFIs for three independent experiments. (**F**) Percentage migration (at 6 h) of input NIH3T3-PZR, -PZRb, and NIH3T3 non-transduced cells on fibronectin, collagen IV, or laminin or in the absence of ECM. PZR enhanced migration on FN compared to non-ECM control (*p* < 0.0001, Student’s *t*-test). (**G**) NIH3T3 or NIH3T3 cells stably expressing PZR or PZRb before (Nil) or after treatment with *PZR2*, *PZR3, Scrambled PZR2 (Scr.PZR2),* or *Scrambled PZR3 (Scr.PZR3)* stained with WM78 followed by a secondary FITC anti-mouse IgG1 were analyzed by flow cytometry. Values are means ± S.E.M. of MFIs for three independent cell clones. (**H**) siRNA knockdown of PZR in NIH3T3-PZR and -PZRb transfectants migrating on fibronectin, showing decreased migration with *PZR2* siRNA compared to *Scr.PZR2* (*p* < 0.001, Student’s *t*-test). Results are means ± S.E.M. of three independent experiments. (**I**) CD29 (red stain) (**a, d,** and **g**) co-localizing with WM78 (green stain) (**b, e,** and **h**) in NIH3T3-PZR cells, with double stained images in **c, f,** and **i.** Similarly, CD29 (**j**) co-localizing (**l**) with WM78 (**k**) in NIH3T3 PZRb cells, but at lower levels. Negative isotype control double staining (mIgG1 and rat IgG2a; **m** and **n**) for NIH3T3-PZR and NIH3T3-PZRb cells, respectively. (**J**) CD49e mAb (red stain) (**a** and **d**) co-localizing with WM78 (green stain) (**b** and **e**) in NIH3T3-PZR cells, with double stained images in (**c**) and (**f**), respectively. Similarly, CD49e mAb (**g**) co-localizing (**h**) with WM78 (**i**) in NIH3T3-PZRb cells at much lower levels. Focal adhesions are also indicated in (**f**) by the arrow. Negative isotype control double staining (mIgG1 and rat IgG2a) in (**j** and **k**) for NIH3T3-PZR and NIH3T3-PZRb cells, respectively. (**K**) CD51 (red stain) (**a**) and WM78 (green) (**b**) staining of NIH3T3-PZR and CD51 (**d**) and WM78 (**e**) of NIH3T3-PZRb cells. Double staining with isotype negative control mAbs (mIgG1 and rat IgG1) is presented in panels (**g**) and (**h**) for NIH3T3-PZR and NIH3T3-PZRb cells, respectively. (**L**) CD49d (red stain) (**a**) and WM78 (green) (**b**) staining in NIH3T3-PZR and CD49d (**d**) and WM78 (**e**) in NIH3T3-PZRb cells, with double staining for isotype negative controls (mIgG1 and rat IgG2a) in (**g**) and (**h**) for NIH3T3-PZR and NIH3T3-PZRb cells, respectively. (**M**) Percentage of co-localization of WM78mAb (PZR) with CD29, CD49d, CD49e, and CD51 in NIH3T3, NIH3T3-PZR, and NIH3T3-PZRb cells. Values are means ± S.E.M. for two independent experiments performed in triplicate.

Since the reported integrin receptors for fibronectin on hBM MSCs include α4β1, α5β1, and αvβ3 integrins [27,28,29], we examined the expression of CD29 (β1), CD49d (α4), CD49e (α5), and CD51 (αv) by flow cytometry on both the NIH3T3 transfectants and on hBM MSCs. As shown in Figure 3A–E, NIH3T3 were strongly and similarly positive for CD49e and CD29 when compared with CD49d or CD51, regardless of whether the cells were transfected with and expressed human PZR or PZRb, or were human PZR and PZRb negative. 

The speed of migration of the human PZR isoform expressing NIH3T3 cell lines on ECM proteins was analyzed (Figure 3). Interestingly, despite similar levels of *PZR* or *PZRb* and integrin expression on NIH3T3 stable transfectants (Figure 3A–E), the human PZR-expressing NIH3T3 cells migrated approximately four-fold faster on fibronectin than the hPZRb-positive or non-transduced NIH3T3 cells (Figure 3F). There was no increase in migration of NIH3T3-PZR cells when laminin or collagen IV were used instead of fibronectin; indeed, migration of these cells on fibronectin was significantly enhanced compared to laminin or collagen IV (*p* < 0.0001 and <0.0005, respectively, using one way ANOVA and Tukey’s HSD post hoc test). This was confirmed by knocking down human PZR and PZRb in the NIH3T3-PZR and NIH3T3-PZRb transfectants (Figure 3G,H) and demonstrating a significant decrease in NIH3T3-PZR cell migration on fibronectin with human *PZR2* siRNA knockdown (*p* < 0.01, *p* < 0.0005, and *p* < 0.001 in comparison with *PZR3*, and *Scr.2,* or *Scr.3* control siRNA knockdown, respectively). 

#### hPZR Clusters with the Fibronectin Receptor VLA-5 during Migration on Fibronectin and Promotes Focal Adhesion Kinase Phosphorylation

Figure 3I–M shows an association between human PZR and CD29 and CD49e molecules, respectively (55% and 48%), but not CD51 or CD49d. Significantly lower levels (2–3-fold less) of human PZRb co-localizing with CD29 and CD49e were detected for NIH3T3-PZRb expressing cells (Figure 3M). This suggests that a specific interaction between the human PZR isoform and the α5β1 integrin could facilitate the migration of human PZR expressing NIH3T3 cells. 

**Figure 4:**
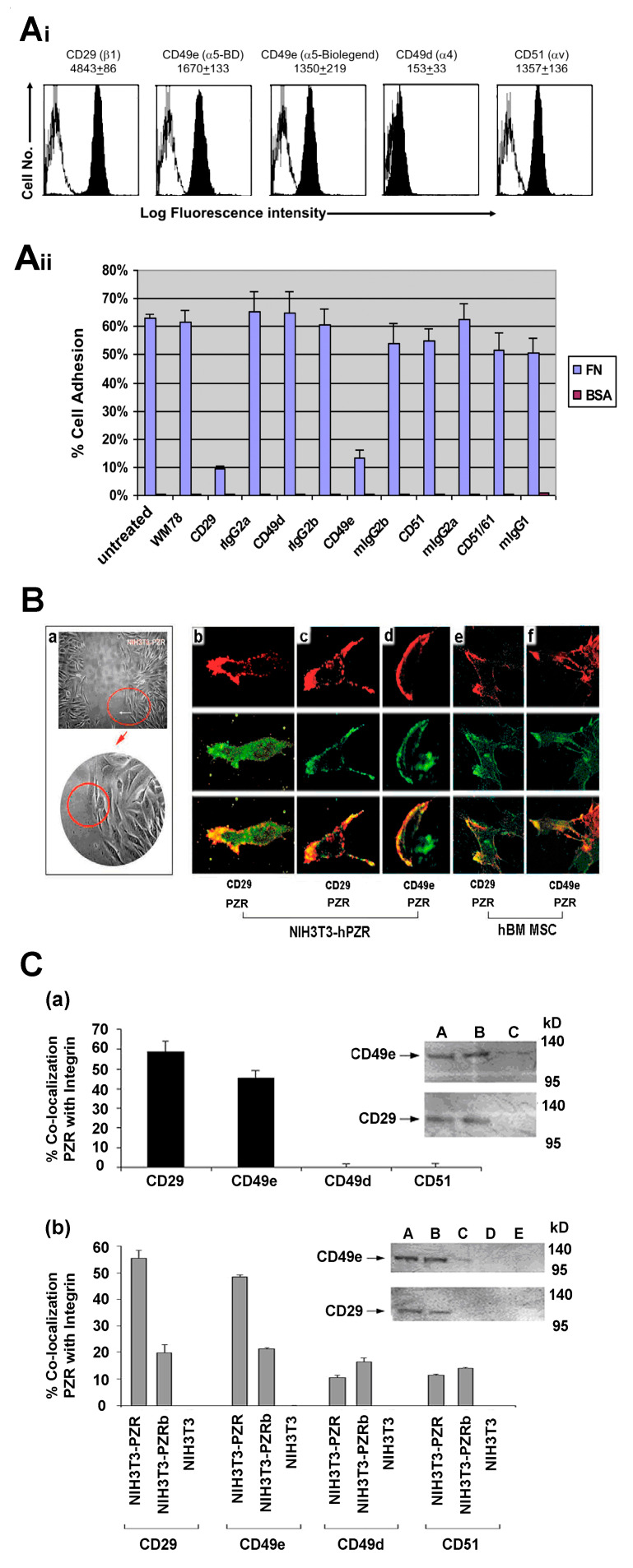
Co-localization of PZR and integrins on migrating NIH3T3 transfectants and hBM MSCs. (**Ai**) Representative FACS histograms of hBM MSCs stained for CD29 (β1 integrin), CD49e (α5 integrin), CD49d (α4 integrin), CD51 (vitronectin receptor), or the relevant isotype control (mIgG1) followed by Alexa-488 goat anti-mIgG1. MFI ± S.E.M. shown above histograms (*n* = 3 independent experiments). Black histograms: integrin staining; white histograms: mIgG1 negative control. (**Aii**) hBM MSCs were untreated or incubated with blocking antibodies against CD29, CD49e, CD49d, CD51, and CD51/61 or the corresponding isotype controls before being allowed to adhere to fibronectin (FN) or the negative control, BSA. Values are means ± S.E.M. for three independent experiments performed in triplicate. (**B**) Migration assay using NIH3T3-hPZR (**a**) with one area analyzed by confocal microscopy circled in red. (**b,c**) NIH3T3-PZR transfectants from the 6 h migratory interface double stained with WM78 (PZR; green stain) and biotin-CD29 (red stain) or (**d**) biotin-CD49e (red stain) plus appropriate secondary fluorescent reagents. hBM MSCs from the 6 h migratory interface double stained with WM78 and Alexa488 goat anti mIgG1 (PZR; green stain), then blocked with mIgG1 and stained with biotinylated (**e**) CD29 (red stain) or (**f**) CD49e (red stain) with streptavidin-conjugated Alexa 546. There is a co-association of PZR with α5 (CD49e) or β1 (CD29) at the leading edge of the migrating cell. **C**(**a**) Quantitation of PZR co-localizing with CD29 and CD49e or CD49d and CD51 in 6 h migrating hBM MSCs. Values are means ± S.E.M. for three independent experiments performed in triplicate. Inset shows co-immuno-precipitation and Western blotting with respective the anti-PZR WM78 mAb and biotin conjugated anti-human CD49e or CD29, and using mIgG1 as the negative control for the immunoprecipitation (i.p.). Lane A: hBM MSC cell lysate, lane B: hBM MSC i.p with anti-PZR, lane C: hBM MSC i.p. with negative control. **C**(**b**) Confocal microscopy of PZR co-localizing with CD29 and CD49e and to a much lesser extent with CD49d and CD51 in NIH3T3-PZR after 6 h of migration. Inset shows co-immunoprecipitation and Western blotting of PZR (WM78 mAb) with biotin anti-mouse CD49e or CD29. mIgG1 was used as negative i.p. control. Lane A: NIH3T3-PZR cell lysate, lane B: NIH3T3-PZR i.p with WM78, lane C: NIH3T3-PZRb i.p. with WM78, lane D: NIH3T3 i.p. with WM78 and lane E: NIH3T3-hPZR i.p. with mIgG1.

We examined the expression of fibronectin receptors on hBM MSCs. In the literature, hBM MSCs are reported to express α1β1, α2β1, α3β1, α4β1, α5β1, α6β1, αvβ1, αvβ3, and α6β4 integrins [27,28,29]. Among these, VLA-4 (α4β1), VLA-5 (α5β1), and VNRα (αvβ3), are known to bind to fibronectin. Therefore, we examined the expression of CD29 (β1), CD49e (α5), CD49d (α4), and CD51 (αv) by flow cytometry. hBM MSCs were highly positive for CD29, CD49e, and CD51 (Figure 4Ai). In contrast, the expression level of CD49d was weak (7.5 ± 4.6% positive). This confirmed that the FN receptors on hBM MSCs include CD29, CD49e, and CD51. Next, we investigated which integrin was responsible for mediating the binding of hBM MSCs to FN (Figure 4Aii). For this, hBM MSCs were incubated with blocking antibodies against CD29, CD49e, CD49d, CD51, and CD51/61 (αvβ3) or the corresponding isotype controls before being allowed to adhere to fibronectin (FN). In the absence of antibody blockade, 62% of hBM MSCs adhered to FN after 30 min. Incubation with isotype controls did not significantly alter this adhesion of hBM MSCs. Among the integrins tested, blocking with CD29 and CD49e resulted in a significantly reduced adhesion of hBM MSCs to FN to 9.30 ± 1.22% for CD29 and 13.09 ± 3.01% for CD49e (*p* < 0.001 for both; Student’s *t*-test). In contrast, blocking of CD49d, CD51, or CD51/61 did not demonstrate a significant difference in adhesion compared to untreated hBM MSCs (*p* > 0.05 for all). These data suggest that, among the FN receptors expressed by hBM MSCs, only CD29 and CD49e are involved in the adhesion to FN.

Using immunofluorescence and confocal microscopy, we next examined whether the CD29/CD49e integrin cluster with the human PZR during migration of hBM MSCs or NIH3T3-PZR and NIH3T3-PZRb transfectants on fibronectin. We double stained NIH3T3-PZR transfectants and hBM MSCs with WM78 plus antibodies to CD29, CD49e, CD49d, or CD51 after 6 h of migration on fibronectin. Significantly higher co-localization of human PZR with CD29 or CD49e when compared with the other integrins was observed for hBM MSCs (Figure 4B,C(a); one way ANOVA *p* < 0.0001; *p*< 0.05 for the CD29 comparison with both CD49d and CD51; *p* < 0.0001 for the CD49e comparison with both CD49d and CD51; Tukey’s HSD post hoc test). These interactions were confirmed for hBM MSCs by immunoprecipitation of the human PZR with the WM78 Mab followed by Western blotting with CD29 or CD49e (Figure 4C(a) lanes A and B). To determine whether both human PZR and PZRb isoforms mediated these interactions, co-localization studies were compared using NIH3T3 transfectants (Figure 4C(b)) and demonstrated a significantly higher co-localization of PZR with CD29 or CD49e than for PZRb (*p* < 0.01 and *p* < 0.001, respectively; one way ANOVA with Tukey’s HSD post hoc test) or with either the CD49d and CD51 integrins. These interactions were confirmed by immunoprecipitation of the human PZR with the WM78 Mab followed by immunoblotting with CD29 or CD49e (Figure 4C(b) lane B). Thus, these studies indicate that human PZR accelerates migration of hBM MSCs on fibronectin via interaction between PZR, the VLA-5 integrin and fibronectin.

**Figure 5:**
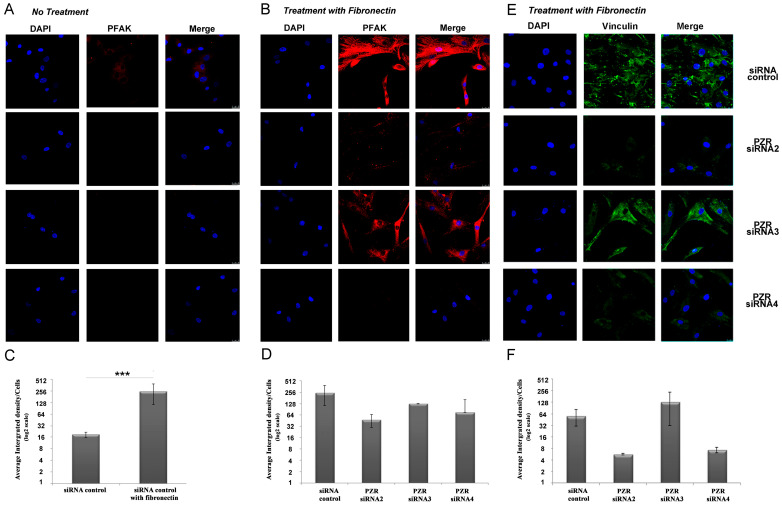
Knockdown of hPZR shows reduced phosphorylation of FAK and reduced vinculin in migrating hBM MSCs. (**A**) Representative confocal microscopy images of PFAK (red) and DAPI (blue) staining in migrating hBM MSCs (**A**) without or (**B**) with fibronectin and treated with *siRNAs* control, *PZR2*, *PZR3*, and *PZR4*. Original magnifications, 63×. (**C**) Quantification of PFAK expression in siRNA control treated hBM MSCs migrating without or fibronectin. The average integrated density of PFAK/the number of cells is shown as means ± S.E.M. per 100 cells counted (*** *p* < 0.001; Student’s *t*-test). (**D**) Quantification of PFAK in hBM MSCs migrating on fibronectin after treatment with *siRNA control, PZR2*, *PZR3,* or *PZR4.* The average integrated density of PFAK/the number of cells is shown as means ± S.E.M. per 100 cells counted. (**E**) Representative confocal microscopy images of hBM MSCs migrating on fibronectin and stained for vinculin (green) and DAPI (blue) after treatment with siRNAs control, *PZR2, PZR3,* or *PZR4*. Original magnifications, 63× (**F**). The average integrated density of vinculin/the number of cells is shown as means ± S.E.M. per 100 cells counted.

To further understand the role of human PZR and human PZRb in regulating cell migration, phosphorylated focal adhesion kinase (PFAK) levels were examined on migrating hBM MSCs after *PZR* and/or *PZRb* knockdown (Figure 5) using fluorescence and confocal microscopy. PFAK expression was significantly enhanced in hBM MSCs after exposure to fibronectin (Figure 5A versus Figure 5B) as exemplified with control siRNA transfected cells (average integrated density/number of cells for siRNA control without fibronectin: 18.7 ± 3.3; siRNA control with fibronectin: 251.7 ± 139.2) (Figure 5C; *p* < 0.001; Student’s *t*-test). In hBM MSCs treated with fibronectin, PFAK was diminished when human PZR was silenced (average integrated density/number of cells for siRNA control: 251.7 ± 139.3, compared to *PZR* 2: 48.1 ± 18.4, and *PZR* 4: 74.4 ± 90.8; ANOVA (*p* < 0.001), and *p ≤* 0.001 and *p* < 0.005, respectively, using Tukey’s HSD post hoc test; Figure 5D). In contrast to human PZRb silenced cells (i.e., those transfected with *PZR3* siRNA; average integrated density/number of cells: 127.1 ± 3.4), almost no short fibers of PFAK could be detected at the cell edges following human PZR (*PZR2* and *PZR4* siRNAs) knockdown (Figure 5D). As vinculin links integrins to the actin cytoskeleton in a pathway involving PFAK, we also examined whether the human PZR modulates vinculin expression. Vinculin expression after fibronectin treatment of hBM MSCs was substantially reduced when human PZR was knocked down with *PZR2* or *PZR4* siRNAs (average integrated density/number of cells for siRNA control: 56.2 ± 25.3, *PZR2* siRNA: 5.7 ± 0.3, *PZR4* siRNA: 7.3 ± 1.1) (Figure 5E,F). This reduction in expression was not observed with *PZR3* siRNA knockdown when compared to *PZR2* and *PZR4* siRNA knockdown (*p* < 0.01 and *p* < 0.05, respectively; one way ANOVA (*p* < 0.01), with Tukey’s HSD post hoc test; Figure 5F). 

These results indicate that human PZR knockdown inhibits the phosphorylation of FAK with a concomitant decrease in the recruitment of vinculin to hBM MSC focal adhesions. 

## 5. Discussion

ECM-mediated cell migration, which occurs in response to specific chemical and mechanical microenvironmental sensing, initially involves cell adhesion to ECM molecules via transmembrane integrin heterodimers, followed by recruitment of intracellular proteins to signaling hubs or adhesomes [30]. These adhesomes are assembled sequentially, first at the plasma membrane as integrin signaling molecules that include FAK, secondly as force transduction molecules including talin and vinculin which link integrins to the actin cytoskeleton, and then as actin regulatory molecules [31,32]. They are disassembled as integrins become inactive, and then reassembled with subsequent ECM-mediated integrin activation, allowing cell migration to proceed [30]. Recently, 60 fibronectin-induced consensus adhesome proteins were identified [30,33,34,35], with a number enriched in hBM MSCs when interacting with fibronectin via the α5β1 integrin [36]. 

In the study presented here, we demonstrated that human PZR, the predominant PZR isoform expressed on hBM MSCs, can most significantly and consistently regulate hBM MSC migration on, but not adhesion to or spreading on, fibronectin, when compared with the other ECM proteins tested, viz. vitronectin, collagens I and IV, and laminin. This is in contrast to NEDD-9 [37], which, in our studies, functioned in regulating hBM MSC adhesion, spreading, and migration to a variety of ECM molecules [22]. With respect to fibronectin-mediated migration, our results identified PZR as a binding partner of α5β1 (VLA-5) integrin upon fibronectin engagement. While this interaction of hBM MSCs with fibronectin enhanced FAK phosphorylation, PZR knockdown reduced FAK phosphorylation and vinculin expression and retarded hBM MSC migration. 

ECM-integrin signaling to the actin cytoskeleton is mediated, in part, through FAK phosphorylation and enhanced talin/vinculin recruitment to adhesomes [30,38,39]. Integrins are also important mechanosensors, which, through FAK activation (phosphorylation), generate stronger cytoskeletal linkages and promote focal adhesion maturation [38,39,40,41]. Following integrin clustering, FAK undergoes autophosphorylation on Y397, which acts as a docking site for SH2 domain proteins (such as c-Src), which subsequently phosphorylate FAK (on Y576 and Y577) to enhance its functions [30,38]. It has been shown that PZR ITIMs can act as substrates for c-Src, c-Fyn, c-Lyn, Csk, and c-Abl [20], and that PZR, as well as other c-Src substrates (including FAK) in its close proximity, can become hyper-tyrosine phosphorylated in Noonan and LEOPARD syndrome murine models [16,17]. While vinculin does not target β1 integrin tails or PFAK, but interacts with such focal adhesion proteins as talin, its loss increases focal-adhesion turnover and random cell migration [42]. Additionally, some of these adhesome molecules (e.g., activated FAK, talin) associate with and maintain the active state of integrins during their endocytic recycling, preparing them for renewed assembly into adhesomes at the leading edge of cells where new adhesions are formed as they migrate, a process termed ‘conformational memory’ [30,38,43,44]. Activated FAK can also translocate to the nucleus, where it regulates cell proliferation and can act as a co-transcription factor regulator [45,46]. 

**Figure 6:**
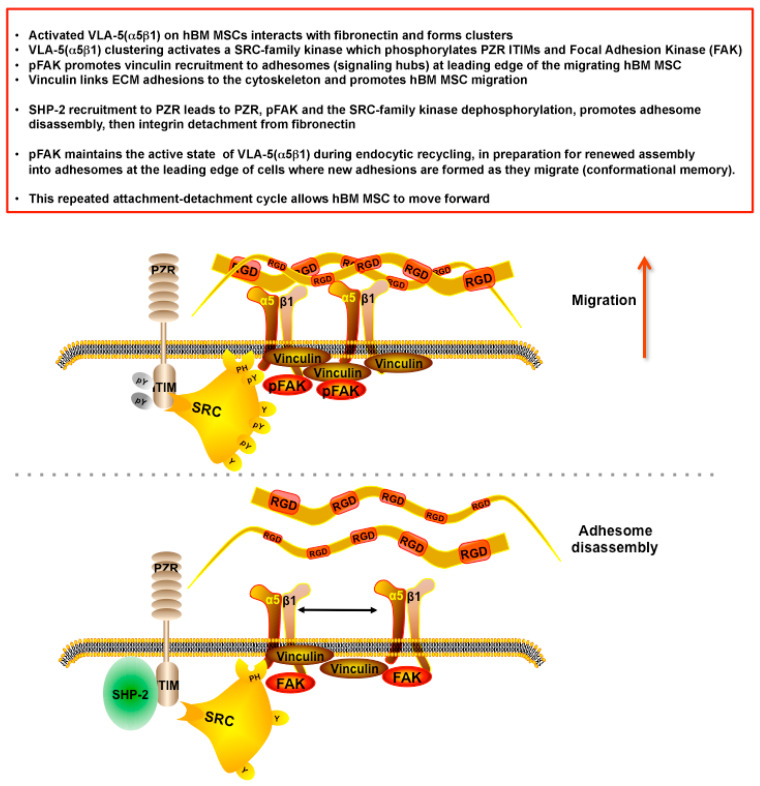
Key results and proposed mechanism of action of PZR in promoting integrin VLA-5(α5β1)-mediated hBM MSC migration on fibronectin. Many proteins are present in adhesomes which are not specified, hence, this diagram provides a simplified mechanism of PZR involvement with specific adhesome proteins that promote hBM MSC migration. An alternative or complementary mechanism would involve endocytic recycling of activated VLA-5(α5β1) by pFAK in preparation for renewed adhesome assembly.

Although further studies are required to fully understand the mechanism of action of PZR, taken together with other published data and as summarized in Figure 6, we speculate that α5β1 integrin clustering, upon engagement with fibronectin, activates a Src-family kinase that rapidly phosphorylates ITIMs in associated PZR molecules in a SHP-2 catalytically independent manner, thereby promoting integrin mediated signaling, phosphorylation of FAK and maintenance of vinculin expression at the leading edge of the migrating cell. PFAK might also be involved in endocytic recycling of α5β1 integrin for the continuation of cell migration. Subsequent recruitment of SHP-2 to PZR would then lead to PZR, PFAK, and the Src-family kinase dephosphorylation, as shown by Marin et al. in another cell type [47], and promote adhesome disassembly prior to integrin detachment from fibronectin so that the cell can move forward as this adhesion-deadhesion cycle repeats itself. Whether promotion of FAK phosphorylation by PZR also leads to increased translocation of PFAK into the nucleus of hBM MSCs thereby regulating cell proliferation and survival has not been addressed and is beyond the scope of these studies. However, it has recently been suggested that MSC cell sheet technology, which preserves MSC-ECM and/or MSC-MSC interactions and hence signaling pathways [48,49,50], may prove more beneficial for certain regenerative medicine applications than the use of chemically or mechanically disrupted MSC preparations. Our studies, which define the role of PZR in VLA-5-mediated hBM MSC migration on fibronectin, are also potentially relevant to deciphering the pathophysiology of Noonan and LEOPARD syndromes. Hyperphosphorylation, rather than dephosphorylation, of PZR mediated by activating or inactivating mutations in SHP-2 [16,17] could inhibit adhesome disassembly, thereby dysregulating hBM MSC migration. Aberrant migration of hBM MSCs, which contain skeletal stem cells that give rise to osteogenic progenitors, may then lead to faulty musculoskeletal tissue formation. 

## Supplementary Materials

The following are available online at https://www.mdpi.com/2073-4409/9/5/1100/s1, Supplementary Figure S1. hBM MSC characterization, Supplementary Figure S2. siRNAs for human PZR and PZRb molecules, Supplementary Figure S3. NEDD9 knockdown in hBM MSC for adhesion and migration assays, Supplementary Figure S4. siRNA knockdown of human PZR and PZRb for spreading and migration assays, Supplementary Figure S5. siRNA knockdown of NEDD9 in hBM MSC prior to the cell spreading assay, Supplementary Figure S6. Representative Western blots for PZR in hBM MSCs in the absence and presence of siRNAs.
