# Supplementary material for: P0-Related Protein Accelerates Human Mesenchymal Stromal Cell Migration by Modulating VLA-5 Interactions with Fibronectin
Source: Cells. 2020 Apr 29;9(5):1100. doi: 10.3390/cells9051100 (PMC7290418; doi:10.3390/cells9051100)
Supplement: Supplementary file 1 [file cells-09-01100-s001.pdf]

Supplementary Materials

NIH3T3 stable transfectants

The murine NIH3T3 mesenchymal cell line was used to generate human PZR and PZRb stable transfectants. In brief, full length human PZR and PZRb cDNAs were cloned into the multiple cloning site (MCS) of the Murine Stem Cell Virus (MSCV-pac retrovirus) vector (Clontech, TaKaRa Bio USA Inc., Mountain View, CA, USA), and viral vectors generated [21]. NIH3T3 cells were cultured overnight in Dulbecco's modified Eagle's medium (DMEM; Sigma-Aldrich Ltd.), and the medium was then replaced with fresh DMEM containing 8 μg/ml polybrene. Cells were transduced independently with the MSCV retroviral supernatant containing the human PZR and PZRb isoforms and positive clones selected for antibiotic resistance using puromycin [21].

Antibodies

FITC- or PE-conjugated CD90, CD146, CD73, CD105, CD45, CD14 and CD34 monoclonal antibodies (mAbs) for hBM MSC characterization have been described in [11,12, 21-25]. Mouse WM78 mAb (mIgG1) to human PZR/PZRb has been described previously [11]. Mouse anti-human NEDD9 mAb (2G9, mIgG1) was purchased from Abcam, Cambridge, England. Murine mAbs to human CD29 (MAR4, mIgG1), human CD49d (9F10, mIgG1), human CD49e (VC5, mIgG1), the equivalent biotin-conjugated mouse anti-human CD29, CD49d, CD49e and CD51 and isotype control mAbs (from BD Pharmingen, BD Biosciences; Molecular Probes, Thermo-Fisher Scientific; Sigma-Aldrich Ltd.; R&D Systems, Abingdon, England; Merck Millipore Ltd., Burlington, MA, USA), as well as biotin-conjugated rat anti-mouse CD29 (9EG7, rat IgG2a), CD49d (9C10, rat IgG2a), CD49e (5H10-27, rat IgG2a), and CD51 (RMV-7, rat IgG1) mAbs, as well as the FITC goat anti-mIgG1, the isotype mIgG1-FITC, mIgG2a-PE and biotinylated rat IgG1 and rat IgG2a controls for these were supplied by BD Pharmingen. Mouse anti-human CD51 (AMF7, mIgG1) mAb was obtained from Serotec Ltd., Kidlington, England. Blocking mAbs were rat anti-human CD29 (Mab13; rat IgG2a) and mouse anti-human CD51/CD61 (23C6; mIgG1) from BD Pharmingen, rat anti-human CD49d (PS/2; rat IgG2b) from Abcam, and mouse anti-human CD49e (NKI-SAM-1, mIgG2b) and mouse anti-human CD51 (NKI-M9, mIgG2a) from Biolegend, San Diego, CA, USA, together with the equivalent isotype control mAbs. Where other non-conjugated mAbs were used, unconjugated mIgG1, mIgG2a, rat IgG2a and rat IgG1 mAbs were used as negative controls (DakoCytomation Ltd. or Southern Biotech, Birmingham, AL, USA). Mouse anti-human vinculin and mouse anti-human beta actin were purchased from Abcam, and alpha-tubulin mAb (B-5-1-2, mIgG1) from Sigma-Aldrich Ltd. Rabbit polyclonal antibodies to human PZR and PFAK (FAK-pY^{397}) were supplied by Cell Signaling, Leiden and Thermo-Fisher Scientific respectively. The secondary Alexa-488 goat anti-mIgG1, Alexa-488 and Alexa-546 streptavidin reagents, Alexa-488 and Alexa-546 goat anti-rat IgG antibodies were from Molecular Probes, Thermo-Fisher Scientific. ImmunoPure peroxidase (HRP)-conjugated goat anti-rabbit and anti-mouse antibodies were purchased from Pierce Biotechnology, Rockford, IL, USA and HRP-Streptavidin was obtained from Chemicon (MerckMillipore Ltd.). The IRDye 800CW goat anti-rabbit IgG and IRDye 680RD donkey or goat anti-mouse IgG secondary antibodies were purchased from Li-COR.

Flow cytometric analysis of cell surface antigens

Adherent cells (hBM MSCs, MEF transfectants, and NIH3T3 transfectants were removed from tissue culture dishes or flasks using accutase (PAA Laboratories GmbH, Pasching, Austria) for 5min at 37 °C, counted and washed in PBS with 0.2% (*wt/vol*) BSA. Cells (up to 1 × 10^5) were then resuspended in 100μl MACS (0.5% (*wt/vol*) BSA in PBS) buffer and incubated with 20% (*vol/vol*) Fc receptor blocking agent (Miltenyi Biotec., Bergisch Gladbach, Germany) on ice for 15min. Fluorescently or biotin conjugated mAbs or isotype-matched control antibodies were added to cells

on ice for 30min. Alternatively, non-conjugated antibodies were added to cells on ice for 30min and the reaction was developed by the addition of FITC-goat anti-mouse IgG1, Alexa-488 goat anti-mouse IgG1 or Alexa-488 or Alexa-546-streptavidin, or Alexa-546 goat anti-rat IgG secondary antibody (2mg/mL; Molecular Probes, Thermo-Fisher Scientific) at 4 °C for 30 min. Cells were washed in MACS buffer, centrifuged at 1,500rpm for 5min and resuspended in 300µL MACS buffer. The ToPro-3 viability dye (Invitrogen Ltd., Thermo Fisher Scientific) was added at a final concentration of 1µM for 5min at RT to exclude non-viable cells. Cells were analyzed on the BD LSR II flow cytometer using the BD FACSDiva software program (both from BD Biosciences) or a Beckman-Coulter EPICS XL-MCL flow cytometer (Ramsey, MN, USA).

Quantitative real time PCR (qRT-PCR)

The human *PZR* and *PZRb* TaqMan primer pairs (Applied Biosystems, Foster City, CA, USA; numbers in the brackets indicate the position of the starting base of the primer in the sequence) were: F *hPZR* (635), 5′-T T A A G C A G G C T C C T C G G A G T -3′, R *hPZR* (742), 5′-C G G A G T G G T C T A A C T G T G C A T A T A T G -3′ and F *hPZRb* (574) 5′-G A A G G A A A A A C T C T A A A C G G G A T T -3′, R hPZRb (688) 5′-G C A T A C A C C A C A G A C T C T G A C T T G T -3′ respectively, as well as dual fluorescent probes *hPZR* (5′-C C C T C C G A C A C T G A G G G T C T T G T A A A G A G T C -3′) and *hPZRb* (5′-A A C T G T G C A T A T A T G A C T G G G C C C C A G T - 3′). *VEGF* served as the positive control and β2-microglobulin as the housekeeping gene. mRNA was isolated using Trizol (Invitrogen Ltd., Thermo Fisher Scientific) and Taqman gene expression assays, with custom predesigned sets of primers and probes for *VEGF* and β2-microglobulin (*B2M*) (Applied Biosystems) or the *hPZR* and *hPZRb* primers above, were carried out as described [11,12,21,22]. In brief, thermocycling conditions used were 2 min at 50 °C, 10 min at 95 °C followed by 40 cycles of 95 °C (15 sec) and 60 °C (1 min). Detection was performed with the ABI Prism 7000 Sequence Detector System. The relative mRNA level in each treatment group was calculated using the relative standard curve method with the control group (β2-microglobulin; B2M) as the calibrator. Analysis of data was carried out using the software provided (ABI Prism 7000 SDS Software).

Lipofectamine transfection method

NIH3T3, NIH3T3-hPZR, NIH-3T3-hPZRb or hBM MSCs cells were transfected using the lipofection method (Lipofectamine 2000, Invitrogen Ltd., Thermo Fisher Scientific). Briefly, one day prior to transfection, cells were plated in 4ml MSCGM growth medium without antibiotics in order to form a 50–70% confluent monolayer at the time of the transfection. For each transfection sample, the appropriate amount of siRNA probe (optimized amount: 2µL from 100µM original stock) was diluted in 500µL of OptiMem I reduced serum medium (Invitrogen Ltd., Thermo Fisher Scientific) without serum. The solution was mixed gently and incubated for 5 min at RT. Five microlitre of Lipofectamine was diluted in 500µL of OptiMemI reduced serum medium (Invitrogen Ltd., Thermo Fisher Scientific) and incubated at RT for 5min. Then, the diluted lipofectamine was combined with the diluted siRNA probe (40nM final concentration), mixed gently and incubated for 20min at RT to allow siRNA: Lipofectamine complexes to form. The mixture was then added to the respective T25 flask by mixing by rocking the flask back and forth. The optimization was performed by monitoring relevant gene expression levels by flow cytometry as described.

Functional studies

Coating surfaces with extracellular matrix proteins

24 well tissue culture plates (Corning Ltd., Loughborough, England) for migration and spreading assays and 96-well dark plates (BD Biosciences) for adhesion assays were coated with 5µg/ml human fibronectin (Sigma-Aldrich Ltd.), human vitronectin (R&D Systems), rat tail collagen I (BD Biosciences), human collagen IV (Chemicon Ltd.), laminin (Sigma-Aldrich Ltd.; Engelbreth-

Holm-Swarm murine sarcoma basement membrane), or 5µg/ml BSA (Sigma-Aldrich Ltd.) at 4°C overnight, the day prior to performing the functional assays.

In vitro hBM MSC adhesion assay

hBM MSCs, untreated, or after siRNA transfection (48h for NEDD9 siRNAs or 72h or PZR siRNAs) or sham transfection, were collected after accutase detachment, washed and resuspended at 1 × 10^6cells/ml in HBSS (PAA Laboratories). The fluorescent dye, 2',7'-bis-(2-carboxyethyl)-5-(and-6)-carboxyfluorescein,acetoxy methyl ester (BCECF-AM, B-1170, Molecular Probes, Thermo-Fisher Scientific) was added to the cells (at a final concentration of 1 µM, at 5µL × 10^6 cells in 1ml medium) at 37 °C for 30mins. Cells were then washed twice, resuspended in serum-free DMEM or complete MSCGM and 2 × 10^4 cells from each treatment added to each well of 96-well plates (BD Biosciences) that had been coated with the relevant ECM molecule or control BSA, and blocked with BSA (10 mg/mL) for 30 mins. at 37 °C After incubation, non-adhering cells were washed between twice, at which stage cell numbers binding to the BSA control well were <10%. The fluorescence of each well was read on a Spectramax Gemini XPS Fluorometer (Molecular Devices, San Jose, CA, USA) with excitation and emission set at 488 and 527 nm, respectively, and a cut off of 515 nm and analysed using SoftMax Pro version 0.1 (Molecular Devices). Each experimental variable was tested in triplicate for each individual experiment and for each of three independent experiments. The percent adhesion was calculated by dividing the fluorescent intensity of the test well by the average fluorescence intensity of the input control.

Immunoprecipitation and immunoblotting

Cell surface biotinylation

Cell surface biotinylation was performed as appropriate using the Fluoreporter cell surface biotinylation kit (Invitrogen Ltd., Thermo Fisher Scientific). Briefly, cells were washed twice with ice-cold PBS, lifted with accutase as above (PAA Laboratories) after incubation for 5min at 37 °C, washed twice with ice cold PBS and 10µL biotin (0.2 mg/mL stock) was added per ml per 1 × 10^7 cells. Cells were incubated with biotin on a rotator at 4 °C for 30min, washed 3 times with ice cold PBS and subjected to cell lysis for subsequent immune precipitation.

Cell lysis and immunoprecipitation

hBM MSCs and NIH3T3 or MEF cell lines without or expressing human PZR or PZRb were cultured on fibronectin, washed in PBS and lysed in RIPA lysis buffer (all reagents from Sigma-Aldrich Ltd. and comprising 50mM Tris-HCl, pH 8.0, 150mM NaCl, 1% (*vol/vol*) IGEPAL-CA-630, 0.5% (*wt/vol*) sodium deoxycholate, 0.1% (*wt/vol*) SDS containing proteinase inhibitor cocktail (1:100)) for 30min on ice. Lysates were centrifuged at 14,000rpm for 30min at 4 °C. Supernatants were collected in a fresh tube. The protein G Sepharose 4 Fast Flow beads (Amersham, GE Healthcare) were washed 3 times in RIPA buffer with the supernatant discarded each time. A 50% slurry was prepared by mixing equal volumes of beads and RIPA buffer and was stored at 4 °C. The cell lysate was pre-cleared with protein G beads by adding 100µL of the 50% slurry to the cell lysate. The suspension was mixed for 2h at 4 °C, followed by centrifugation at 12,000rpm for 1min and the supernatant was retained. Five µg of WM78 mAb, rabbit anti-human PZR, or negative control antibodies were added to the supernatant and mixed at 4°C overnight. The protein complexes were isolated by incubation with 50µL protein G Sepharose 50% slurry. This was mixed at 4 °C for 2h and centrifuged at 12,000rpm for 1min and the pellet retained. The pellet was subsequently washed 3 times with RIPA buffer with the final pellet being resuspended in 50µL sample buffer. This was heated at 95 °C for 5min and centrifuged at 12,000rpm to remove the beads, which pellet, and the supernatant was retained for protein analysis. All other cell lysis (e.g. for NEDD9 and its alpha-tubulin control Western blots) was carried out using treatment buffer (75mM Tris-HCL, pH6.8 (Sigma-Aldich Ltd.), 3.8% (*wt/vol*) SDS (Sigma-Aldrich Ltd.), 4M Urea (*wt/vol*)

(Sigma-Aldich Ltd.), β-mercaptoethanol (BME, Sigma-Aldich Ltd.) and 20% (*vol/vol*) glycerol (Sigma-Aldrich Ltd.). Subsequent Western blots were carried out as described in the Materials and Methods.

Bio-Rad Dc Protein Assay

Protein was measured using the Biorad Dc Protein Assay. A BSA stock of 2 mg/ml was used to prepare solutions for the standard curve. Twenty μL of reagent S (Biorad Laboratories Ltd, Hercules, CA, USA) was added to each ml of reagent A (Biorad Laboratories Ltd). Five μL of standards or samples to be tested were pipetted in triplicate into a microtiter plate with 5μL PBS as the blank. Then, 25μL reagent of A and 200Ml of reagent B (Biorad Laboratories Ltd) were added to each well. The plate was briefly agitated and read at 655nm in the BioRad Model 450 microplate reader (Biorad Laboratories Ltd). Program MPMIII was used for the analysis of the results.

Confocal microscopy co-localization studies

One to 5 × 10^5 hBM MSCs or NIH3T3 transfectants/well were cultured overnight in four chamber polystyrene vessel tissue culture treated glass slides (Becton-Dickinson) coated with 20μg/mL fibronectin overnight at 4 °C. All incubations are carried out using media at 4 °C unless stated otherwise. Cells were washed by immersing gently in PBS and 0.1% sodium azide buffer (wash buffer) and slides dried off except liquid in the well. Fifty microlitres (10μg/mL) primary antibody or isotype specific controls were added to each well. Slides were incubated for 30min-1hour and washed 3 times in PBS with 0.2% (*wt/vol*) BSA. Fifty microlitres of (10μg/mL) streptavidin-conjugated Alexa 546 anti-rat IgG2a, anti-rat IgG1, or Alexa-488 goat anti-mIgG1 (Molecular Probes, Thermo-Fisher Scientific) secondary antibody, diluted 1/1000 in PBS with 0.2% BSA, were added to the slides for 60min. Cells were washed in PBS and then fixed with 50μL 3% paraformaldehyde for 10min at RT, washed in PBS and mounted with fluorescent mounting medium (DakoCytomation Ltd.). Images were acquired using an automated Zeiss 510 confocal microscope (Carl Zeiss Microimaging Inc.) fitted with HeNe543, HeNe 633 and argon 488 lasers. The fluorescent images were exported and further processed using Imaris 3.3 software (Bitplane AG). The percentage of co-localization of the proteins of interest was determined for antibody stained cells at the interface with the scratch in the wound healing assay using the Imaris co-localization module of Imaris software. More specifically, image stacks for each antibody stained individual cell were imported into the Imaris image processing software, which determined electronically the area of co-labeling versus single antibody labeling of the proteins of interest. The percent of protein co-localization was then determined by measuring the areas jointly stained by both antibodies (overlapping areas) divided by the total areas stained by either antibody or both antibodies together (total area) for each individual cell and then multiplied by 100 to give the percentage of co-localization. At least 100 cells were examined in each of three experiments.

Supplementary Results

NEDD9 as a positive control for the adhesion, migration and spreading assays.

NEDD9 (Neural precursor cell expressed, Developmentally Down-regulated 9; also known as HEF1 [Human Enhancer of Filamentation 1] and Cas-L [Crk-associated substrate-related protein, lymphocyte type] is a scaffolding, adaptor protein, composed of 824 amino acids, which contains several protein-protein interaction domains [37 and references therein]. NEDD9 contains i) an N-terminal SH3 domain (amino acids 10–65), which is responsible for binding to proteins containing a poly-proline motif, ii) a substrate domain encompassing a large number of SH2 binding sites (amino acids 90–350) and which confers interaction with proteins that contain SH2 domains, iii) a serine rich region (amino acids 350–650) and finally iv) a C-terminal domain (amino acids 650–834), which is thought to be responsible for its interaction with helix-loop-helix (HLH) proteins, thus mediating its homo- or hetero-dimerisation [37 and references therein]. It interacts with other

integrin effector proteins, like Crk, Nck and SHP-2, upon adhesion to ECM through integrins. NEDD9 has been reported to promote integrin activation during cell adhesion, cell spreading and the ''migratory switch'', stabilizing focal adhesions and increasing adhesion force to the ECM [37 and references therein].

We showed that NEDD9 is expressed on hBM MSCs (Supplementary Figure S3). NEDD9 was used in siRNA knock-down studies as a positive control for our PZR adhesion, spreading and migration studies. Two NEDD9 siRNAs, a NEDD9-1 pool and NEDD9-2, were used to knockdown expression of the NEDD9 protein (Supplementary Figure S3). These NEDD9 siRNAs were introduced into hBM MSC by Lipofectamine transfection for each functional assay described and the effect of knockdown detected by Western blotting.

Knockdown of NEDD9 levels reduces hBM MSC adhesion on ECM substrates

For the siRNA transfection, hBM MSCs were plated the day before at 40–60% confluency in complete MSCGM without antibiotics. The next day, cells were either sham transfected or individually transfected with control, NEDD9-1 and NEDD9-2 siRNAs. Following transfection, NEDD9 levels were significantly reduced, compared to the siRNA control levels (Supplementary Figure S3), with the NEDD9 siRNAs reducing the level of each MWt band especially that at 105kD. The control siRNA did not change the levels of NEDD9 compared to the sham transfected cells (p = 0.9999, Tukey's HSD post hoc test). Notably, cell numbers or viabilities were not significantly affected by any of the treatments. hBM MSC were fluorescently labeled and allowed to adhere to the ECM substrates fibronectin, vitronectin, collagen I, collagen IV and laminin (Figure 2G). Cell adhesion was calculated by expressing the test well fluorescence value as a percentage of fluorescence readout for the input control. On fibronectin, significant changes were observed in the adhesion of cells treated with NEDD9 siRNAs compared to the control siRNA treated cells. More specifically, only 39.1±7.3% cells treated with NEDD9-1 siRNA and 40.7 ± 5.6% (both $p < 0.05$, Tukey's HSD post hoc test) cells treated with NEDD9-2 siRNA adhered to fibronectin, whereas 61.1 ± 8.3% of the siRNA control treated cells adhered to fibronectin within 30min of the adhesion assay. No statistically significant changes were observed between sham transfected cells compared to control siRNA treated cells (p = 0.9841, Tukey's HSD post hoc test). On vitronectin, a significant reduction of adhesion took place when cells were transfected either with NEDD9-1 siRNA (17.7% less cells; $p < 0.05$, Tukey's HSD post hoc test) or with NEDD9-2 siRNA (20.7% less cells; $p < 0.01$) compared to control siRNA treated cells. Sham transfected cells adhered to vitronectin in a similar manner as the control siRNA treated cells. On collagen I, lowering NEDD9 expression with NEDD9-1 and NEDD9-2 siRNAs (both $p < 0.01$, Tukey's HSD post hoc test) significantly decreased the adhesive ability of cells compared to the control siRNA treated cells. Slightly more control siRNA treated cells than sham transfected cells were observed to adhere on collagen I, however this was not statistically significant (p = 0.6006, Tukey's HSD post hoc test). On collagen IV, both NEDD9 siRNAs reduced the adhesion of hBM MSCs when compared to the control siRNA treated cells. However only treatment with NEDD9-1, but not NEDD9-2, siRNA reached statistical significance ($p < 0.05$ and p = 0.0671 respectively, Tukey's HSD post hoc test). A minor, but non-significant, difference was observed in adhesion between sham transfected and control siRNA treated cells (p = 0.9481, Tukey's HSD post hoc test). Finally, on laminin, the overall levels of hBM MSC adhesion were low. Cellular adhesion was significantly altered using both NEDD9 siRNAs, with only 14.8 ± 1.3% NEDD9-1 siRNA treated cells and 13.8 ± 1.3% NEDD9-2 siRNA treated cells being able to adhere (both $p < 0.01$, Tukey's HSD post hoc test). No significant differences were detected in the adhesion of sham transfected cells compared to siRNA control treated cells on laminin (p = 0.4084, Tukey's HSD post hoc test).

NEDD9, but not PZR, regulates hBM MSC spreading on ECM substrates

On fibronectin, *PZR2* siRNA-treated cells exhibited delayed spreading after adhesion ($p < 0.05$, Tukey's HSD post hoc test), whereas *PZR3*, *PZR4* and *PZR1* (p = 0.8559, p = 0.9949 and p = 1.0000

respectively, Tukey's HSD post hoc test) siRNA treated cells spread to the same extent as the control siRNA transfected cells (Figure 2E; Supplementary Figure S4). On vitronectin, less spreading cells appeared in the *PZR2* siRNA ($p < 0.05$, Tukey's HSD post hoc test) treated cultures (27%) than in the control siRNA treated cultures (39.5%) within 60 min after plating. However, the introduction of *PZR4* and *PZR1* siRNAs did not significantly decrease the numbers of spreading cells ($p = 0.9716$ and $p = 0.9969$, Tukey's HSD post hoc test, respectively; Figure 2E). On collagen I, *PZR2*, *PZR4* and *PZR1* siRNAs did not have a significant effect on cell spreading on this particular extracellular matrix ($p = 0.0932$, $p = 0.9994$ and $p = 0.9843$ respectively, Tukey's HSD post hoc test; Figure 2E). On collagen IV, a reduction by 20% was observed in cell spreading when cells were treated with *PZR2* siRNA ($p < 0.01$, Tukey's HSD post hoc test) compared to the control siRNA treated cells. No significant changes were observed in cell spreading for cells treated with *PZR3, PZR4* or *PZR1* siRNAs compared to cells transfected with control siRNA ($p = 0.5723$, $p = 0.9872$ and $p = 0.8913$ respectively, Tukey's HSD post hoc test; Figure 2E). Finally, cells showed a trend for decreased spreading on laminin compared to other ECM proteins and this was also observed in their ability to adhere to this extracellular matrix protein. However, although less cells were able to spread when they were treated with *PZR2* siRNA (5%) compared to control siRNA (13.9%) treated cells, this was not significant ($p = 0.4790$, Tukey's HSD post hoc test). *PZR4* and *PZR1* siRNAs also did not significantly alter the percentage of cells spreading on laminin (Figure 2E).

Loss of function assays were also used to determine the role of NEDD9 in regulating hBM MSC spreading on selected ECM proteins. As for adhesion, two siRNAs, NEDD9-1 and NEDD9-2, were used to knockdown expression of the NEDD9 protein prior to introducing cells into the spreading assays. Following transfection, NEDD9 levels were significantly reduced, compared to the siRNA control levels (Supplementary Figure S5), with the NEDD9 siRNAs reducing the level of each MWt band especially that at 105kD. NEDD9 expression levels were not significantly different between sham transfected and control siRNA treated cells ($p = 0.9989$, Tukey's HSD post hoc test). Cell numbers and viability were not affected by any treatment NEDD9 regulates hBM MSC spreading on ECM substrates (Supplementary Figure S5). On fibronectin, NEDD9-1 siRNA treated cells exhibited delayed spreading after adhesion (18.7±3.4%; $p < 0.01$, Tukey's HSD post hoc test) compared to control siRNA transfected cells (37.3±4.9%) (Figure 2H). NEDD9-2 siRNA treated cells also exhibited a reduced ability to spread to fibronectin (23.3 ± 5.2%; $p < 0.05$, Tukey's HSD post hoc test). Sham transfected cells spread on fibronectin to the same extent as the control siRNA treated cells ($p = 1.000$, Tukey's HSD post hoc test; Figure 2H). On vitronectin, less spreading cells appeared in the NEDD9-1 siRNA (32.4 ± 4.0%; $p < 0.05$) and in the NEDD9-2 siRNA (35.3 ± 5.9%; $p = 0.0913$, both Tukey's HSD post hoc test) treated cells than in the control siRNA treated cells (46.9 ± 2.6%) within 60 min after plating (Figure 2H). On collagen I, both NEDD9 siRNAs significantly inhibited cell spreading compared to control siRNA treated cells (38.1 ± 4.4%), as only 25.6 ± 5.3% and 23.0 ± 3.4% (both $p < 0.05$, Tukey's HSD post hoc test) of the respective cells spread after 60min (Figure 2H). On collagen IV, introduction of NEDD9-1 and NEDD9-2 siRNAs resulted in less cells spreading on this substrate (20.4 ± 1.6%; $p < 0.01$ and 22.7 ± 4.9% respectively; both $p < 0.05$, Tukey's HSD post hoc test) compared to the control siRNA treated cells (37.2 ± 2.9%). No significant changes were observed in cell spreading between the sham transfected cells and the control siRNA treated cells ($p = 0.8320$, Tukey's HSD post hoc test; Figure 2H). Finally, hBM MSCs showed overall reduced ability to spread on laminin. On this matrix, less cells were able to spread when they were treated with NEDD9-1 siRNA (7.2 ± 3.2%) and NEDD9-2 siRNA (6.3 ± 6.9%) compared to control siRNA (12.5 ± 3%) treated cells, but this was not significant ($p = 0.5496$ and $p = 0.4291$ respectively; Tukey's HSD post hoc test; Figure 2H). Sham transfected cells on laminin spread to the same extend as the control siRNA treated cells did ($p = 0.9310$, Tukey's HSD post hoc test; Figure 2H).

Knockdown of NEDD9 levels reduces hBM MSC migration on ECM substrates

The two siRNA duplexes, NEDD9-1 and NEDD9-2 were used to knockdown expression of the NEDD9 protein, prior to the performance of the migration assays. Following transfection, NEDD9

levels were significantly reduced, compared to the siRNA control levels (Supplementary Figure S3), with the NEDD9 siRNAs reducing the level of each MWt band, especially that at 105kD. The control siRNA did not change the levels of NEDD9 compared to the sham transfected cells ($p = 0.53$, Student's *t*-Test). Cell numbers were not affected by introduction of NEDD9 siRNAs into the cells (Supplementary Figure S3). Following a reduction of NEDD9 protein levels using siRNAs, a migration assay was performed. On fibronectin, migration of control siRNA treated hBM MSC was not different to sham transfected cells ($p = 0.9481$, Tukey's HSD post hoc test; Figure 2I). However, NEDD9-1 and NEDD9-2 siRNAs significantly inhibited the migratory ability of hBM MSCs, by 42.8 ± 1.9% ($p < 0.0001$) and 47.4 ± 3.2% ($p < 0.0001$, both Tukey's HSD post hoc test) respectively, compared to the normalised control siRNA treated cells (Figure 2I). On vitronectin, sham transfected cells showed a tendency to migrate more than the control siRNA treated hBM MSCs, nevertheless this was not statistically significant ($p = 0.9359$, Tukey's HSD post hoc test; Figure 2I). In contrast, hBM MSC treated with the NEDD9-1 and NEDD9-2 siRNAs showed a reduced ability to migrate on vitronectin, with migration inhibited by 38.7 ± 3.3% ($p < 0.0001$) and 33.3 ± 4% ($p < 0.0001$, both Tukey's HSD post hoc test) respectively, compared to control siRNA treated cells. On collagen I (Figure 2I), hBM MSC treated with the NEDD9-1 and NEDD9-2 siRNAs showed a significantly reduced ability to migrate on collagen I, with migration inhibited by 46.2±6.7% and 39.4 ± 13.2% ($p < 0.0001$ and $p < 0.0005$, Tukey's HSD post hoc test) respectively, compared to control siRNA treated cells. On collagen IV, control siRNA treated hBM MSC migration was not different to sham transfected cells ($p = 0.4557$, Tukey's HSD post hoc test; Figure 2I). In contrast, hBM MSC treated with the NEDD9-1 and NEDD9-2 siRNAs showed a reduced ability to migrate on collagen IV, with migration inhibited by 27.3 ± 6.4% ($p < 0.0005$) and 29.8 ± 4.1% ($p < 0.0001$, both Tukey's HSD post hoc test) respectively, compared to control siRNA treated cells (Figure 2I). Finally, on laminin coated plates, control siRNA treated hBM MSC migration was not different to sham transfected cells ($p = 0.9982$, Tukey's HSD post hoc test; Figure 2I). In contrast, hBM MSC treated with the NEDD9-1 and NEDD9-2 siRNAs showed a reduced ability to migrate on laminin, with migration inhibited by 36.2 ± 9.5% ($p < 0.005$) and 28.2 ± 11.9% ($p < 0.01$, both Tukey's HSD post hoc test) respectively, compared to control siRNA treated cells (Figure 2I).

Supplementary Figure Legends

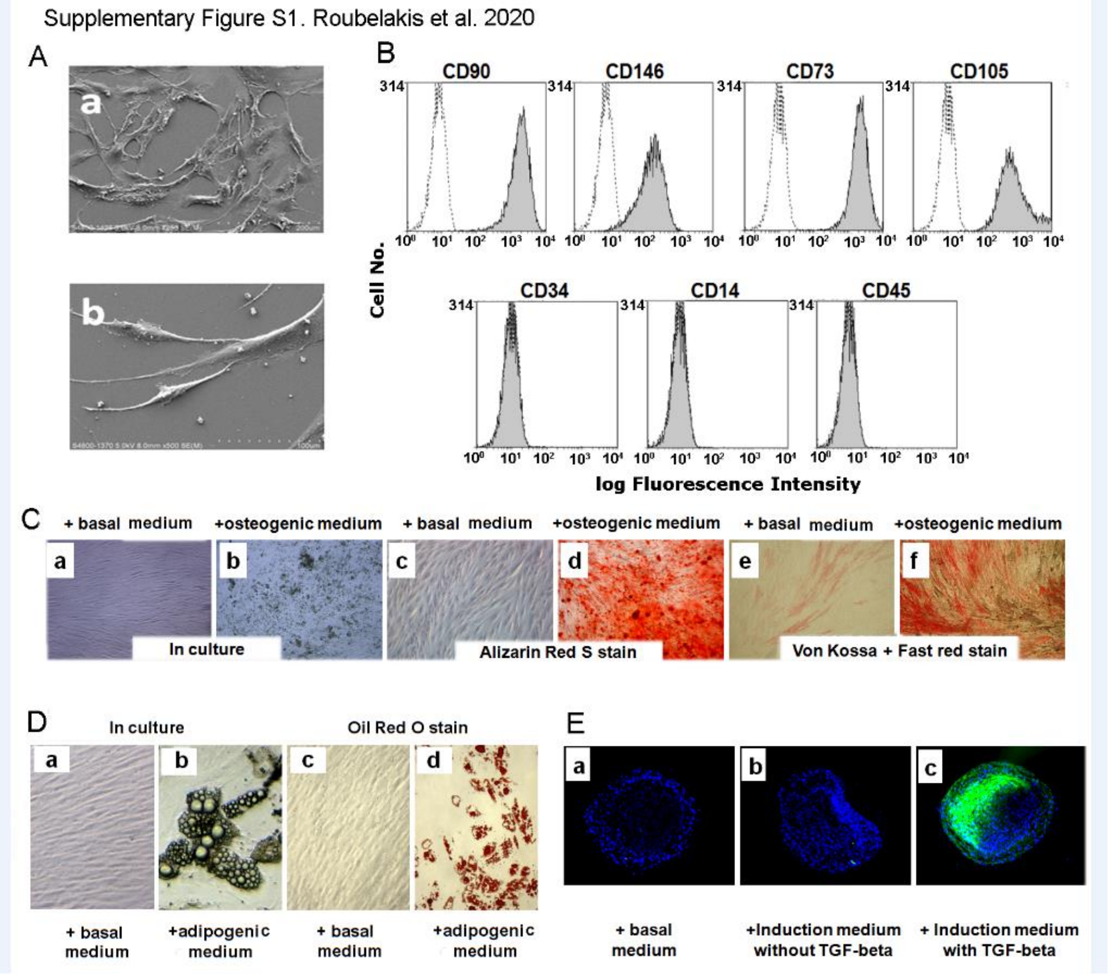

Supplementary Figure S1. hBM MSC characterization: A) (a, b) Scanning electronic microscopy illustrating fibroblastic morphology of hBM MSCs. **B**) Flow cytometric analysis of hBM MSCs for surface marker expression. The white histogram represents the isotype control, the grey histogram represents hBM MSCs stained with the corresponding antibody. **C**) Osteogenic induction (b,d,f) of hBM MSCs compared to cells in basal medium (a, c, e). Cells in culture: a, b; cells stained with Alizarin Red S: c, d, or with Von Kossa + Fast Red stain: e, f. **D**) Adipogenic induction (b,d) of hBM MSCs compared to cells in basal medium (a, c). Cells in culture: a, b; cells with Oil Red stain: c, d. **E**) Chondrogenic induction of hBM MSCs compared to cells in basal medium. Cells in basal medium: a, cells in induction medium without TGF-β: b, and cells in induction medium with TGF-β: c.

Supplementary Figure S2. Roubelakis et al. 2020

A

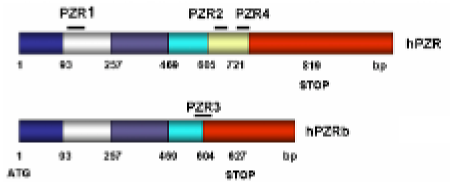

siRNA	Specificity of hPZR and hPZRb siRNAs
PZR1	hPZR and hPZRb
PZR2	hPZR
PZR3	hPZRb
PZR4	hPZR

B

C

D

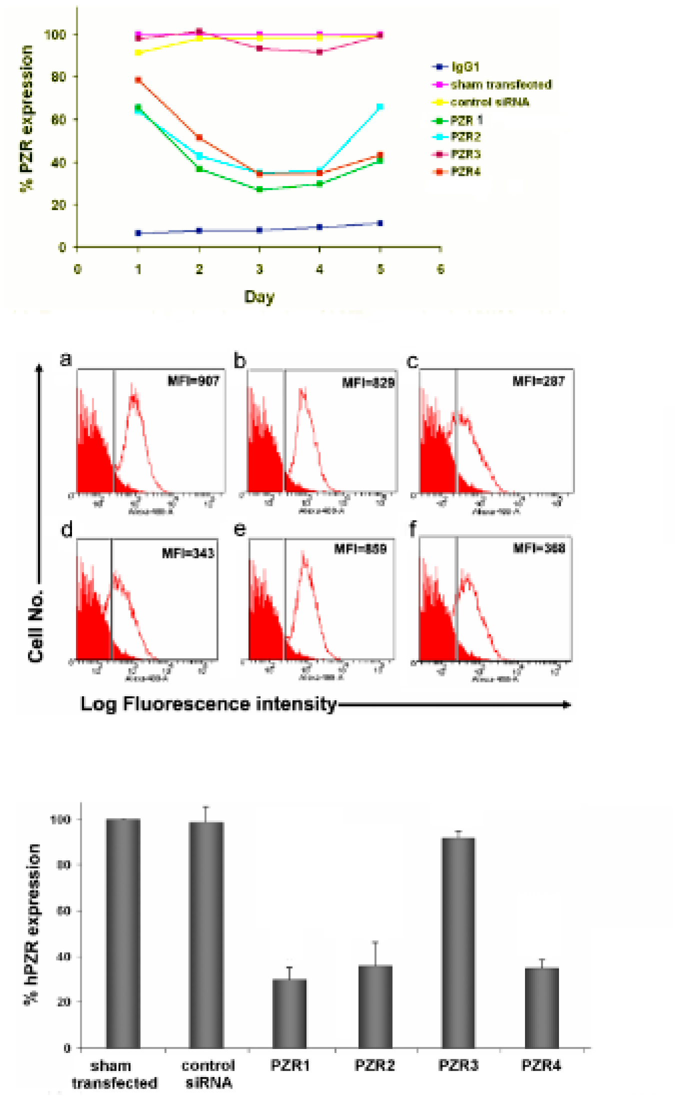

E

siRNA	siRNA specificity	Cell Count (x10^5) after siRNA Treatment	% Cell viability after siRNA Treatment
Nil	Nil	2.94±0.2	98.7±1.1
control siRNA	Nil	2.90±0.3	97.8±1.4
PZR1	hPZR + hPZRb	2.82±0.5	96.4±1.2
PZR2	hPZR	2.76±0.4	97.3±1.9
PZR3	hPZRb	2.70±0.6	96.3±2.2
PZR4	hPZR	2.86±0.2	98.0±0.5

Supplementary Figure S2. siRNAs for human PZR and PZRb molecules: **A**). Schematic diagram of the localization of human *PZR* and *PZRb* siRNAs on the human *PZR* and *PZRb* mRNA. The *PZR1* siRNA probe was designed for the extracellular common region of human *PZR* and *PZRb* (exon 2). siRNA probes *PZR2* and *PZR4* are unique for *PZR* and were designed to exon 5. *PZR3* was designed for the junction of exons 4 and 6 and is unique for *PZRb*. **B**) Time course of knocking down human *PZR* and *PZRb* after siRNA treatment of hBM MSCs as assessed by flow cytometric analysis using the WM78 mAb and normalized to 100% for the untreated control cells. The blue line is the mIgG1 isotype control. siRNAs *PZR1* (light green line), *PZR2* (light blue line), *PZR3* (purple line), and *PZR4* (brown line) were used. Non-transfected hBM MSCs stained with the WM78 mAb are also indicated (pink line). **C**) Flow cytometric plots before and after hBM MSCs were treated with siRNAs to human PZR and PZRb. Representative histograms showing staining with WM78 mAb to hBM MSCs of sham transfected (a) or control (b), *PZR1* (c), *PZR2* (d), *PZR3* (e) and *PZR4* (f) siRNAs and developed with Alexa-488 goat anti-mIgG1 (white profile). Isotype matched mIgG1 negative control mAb staining was used together with an Alexa 488 goat anti-mIgG1 secondary antibody (red profile). The median fluorescence intensity (MFI) of the hBM MSCs stained with WM78 mAb is shown on each histogram. Cells were analyzed on a BD LSR II flow cytometer. **D**) Histograms showing the relative knockdown of human PZR expression, after staining of hBM MSCs treated with siRNAs against hPZR and hPZRb with the WM78 mAb, followed by Alexa-488 goat anti-mouse IgG1. These were compared to sham transfected cells, which were normalized to 100%. Values represent mean ± S.E.M. (n = 3 independent experiments using 3 different batches of hBM MSCs). E) Effects of siRNA treatments on proliferation and viability of hBM MSCs. At day one, 2×10^5 hBM MSCs were transfected with the respective siRNAs and the cell count using a hemocytometer and cell viability were determined after 3 days. Values represent mean ± S.E.M. for 3 independent experiments using 3 different batches of hBM MSCs. Viability was defined as negative staining for the ToPro-3 dye.

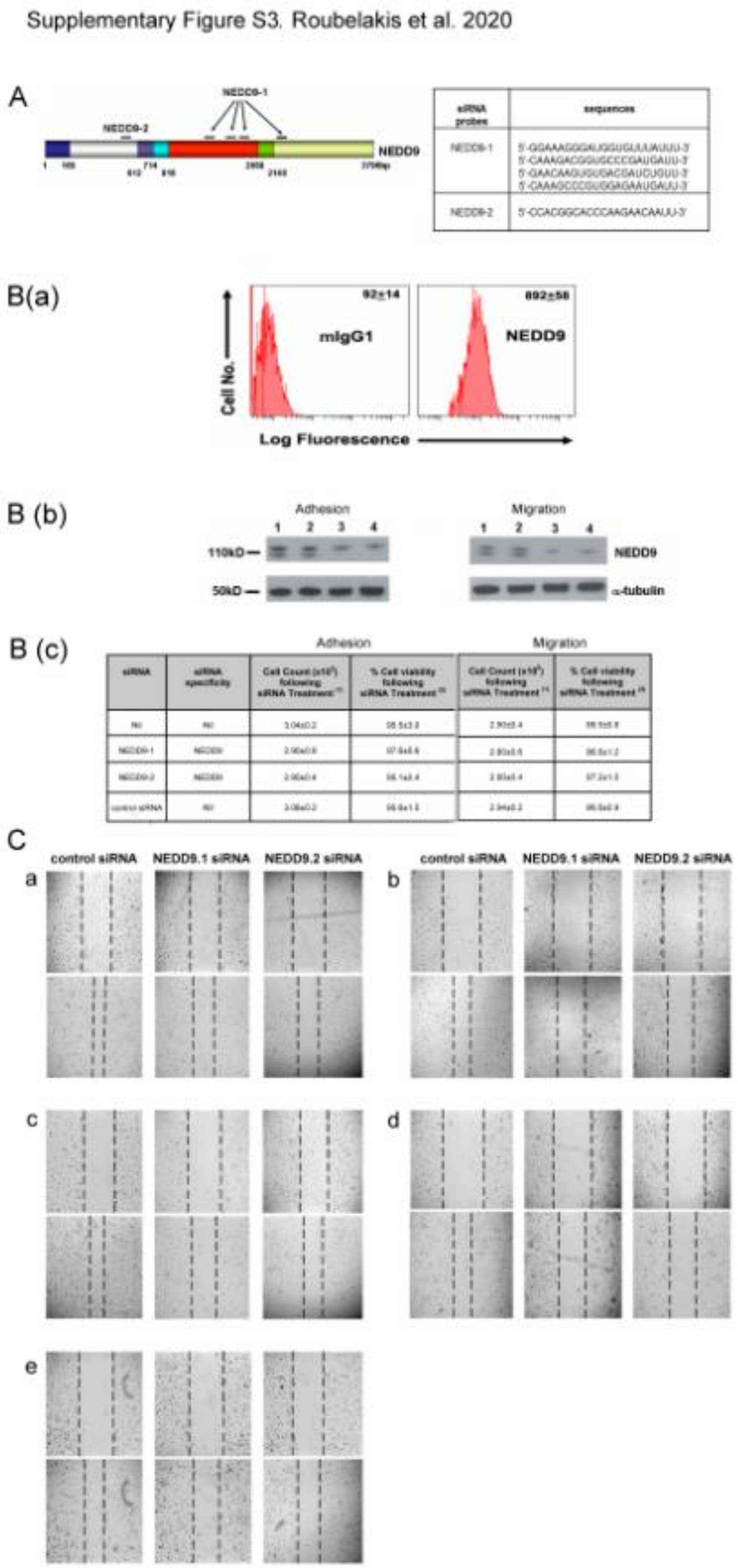

Supplementary Figure S3. NEDD9 knockdown in hBM MSC for adhesion and migration assays: **A**) Schematic diagram of NEDD9 and the targets against which the siRNA probes were designed.

NEDD9-1 siRNA is a SMARTpool siRNA, a collection consisting of four individual siRNA duplexes all targeting specifically NEDD9 (3 target exon 5 and one targets exon 7), whereas NEDD9-2 is an individual siRNA duplex against exon 2. **B**) (a) Representative FACS histogram of cultured hBM MSCs stained with the anti-NEDD9 mAb, 2G9. Cells were grown in T75 flasks with complete MSCGM containing 10% FCS under normoxic conditions. Cells were permeabilized and stained with either the isotype matched mIgG1 negative control (left histogram) or the anti-NEDD9 mAb 2G9 (right histogram), followed by Alexa488-goat anti-mouse IgG1 secondary antibody and analyzed on the BD LSR II flow cytometer. The median fluorescence intensity (MFI) is shown on each histogram, where values are means±S.E.M. of 3 independent experiments using 3 different hBM MSC batches. (b) Representative Western blots for NEDD9 in hBM MSC cell lysates (15μg) from sham transfected (lane 1) or transfected with control (lane 2) or NEDD9-1 and NEDD9-2 siRNAs (lanes 3 and 4 respectively) cells, using the mouse anti-human NEDD9 mAb (2G9) or α-tubulin as a loading control plus HRP-goat anti-mIgG1 (n = 3 independent experiments using 3 different batches of hBM MSCs). Two bands were detected in the sham transfected cells. The lower band had an apparent molecular weight (MWt) of 105kD. The 115kD band is thought to be serine/threonine phosphorylated and both bands are thought to be tyrosine phosphorylated. Left blots were those for hBM MSC adhesion assays and right blots those for migration assays. c) At day one, $2x10^5$ hBM MSCs were transfected with the respective siRNAs and the cells counted using a hemocytometer and cell viability was determined after 2 days. Values represent mean±S.E.M. for 3 independent experiments using 3 different batches of hBM MSCs. Viability was defined as negative staining for the ToPro-3 dye. **C**) Phase contrast images of migration assays of NEDD9-1, NEDD9-2, control siRNA treated or sham transfected hBM MSCs on ECM-coated dishes at 0 (upper panel) and 20h (bottom panel) after initiating the migration assay. Cells were plated in complete medium at a confluent density and scratched with a micropipette tip. ECM molecules used were (a) FN, (b) VN, (c) COL-I, (d) COL-IV and (e) LN.

Supplementary Figure S4. Roubelakis et al. 2020

A

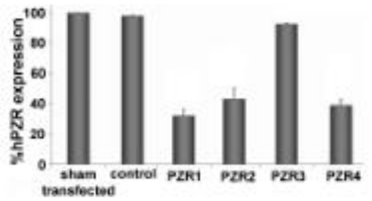

D

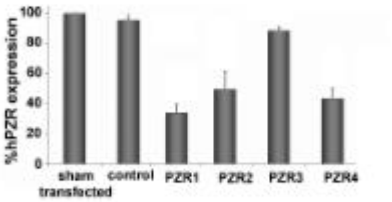

B

siRNA	siRNA specificity	Cell Count $(x10^{4})$ following siRNA Treatment	% Cell viability following siRNA Treatment
Nil	Nil	2.98±0.6	96±1.3
PZR1	hPZR	2.78±0.1	97±0.9
PZR2	hPZR	2.74±0.4	95.6±2.1
PZR3	hPZRb	2.90±0.3	96.3±2.4
PZR4	hPZR	2.92±0.3	97.3±0.7
control siRNA	Nil	2.84±0.5	96.9±1.8

E

siRNA	siRNA specificity	Cell Count $(x10^{4})$ following siRNA Treatment	% Cell viability following siRNA Treatment
Nil	Nil	3.06±0.4	98.2±0.6
PZR1	hPZR	2.68±0.3	95.1±3.5
PZR2	hPZR	2.84±0.4	98.4±1.4
PZR3	hPZRb	2.80±0.6	97.9±1.7
PZR4	hPZR	2.62±0.6	96.6±2.1
control siRNA	Nil	2.96±0.2	96.5±2.4

C

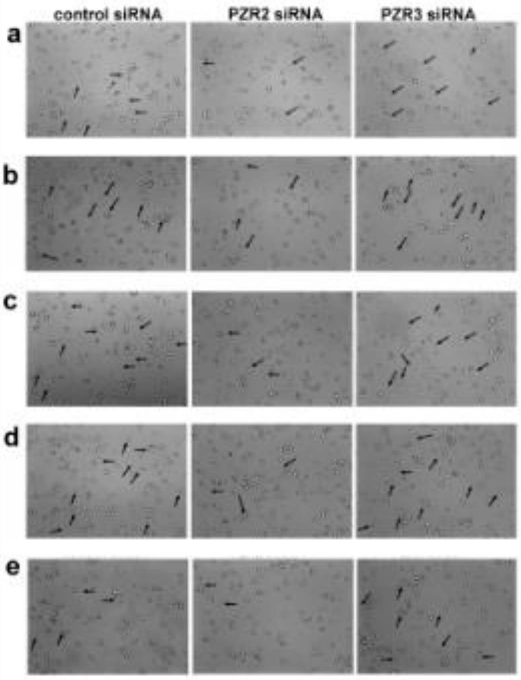

F

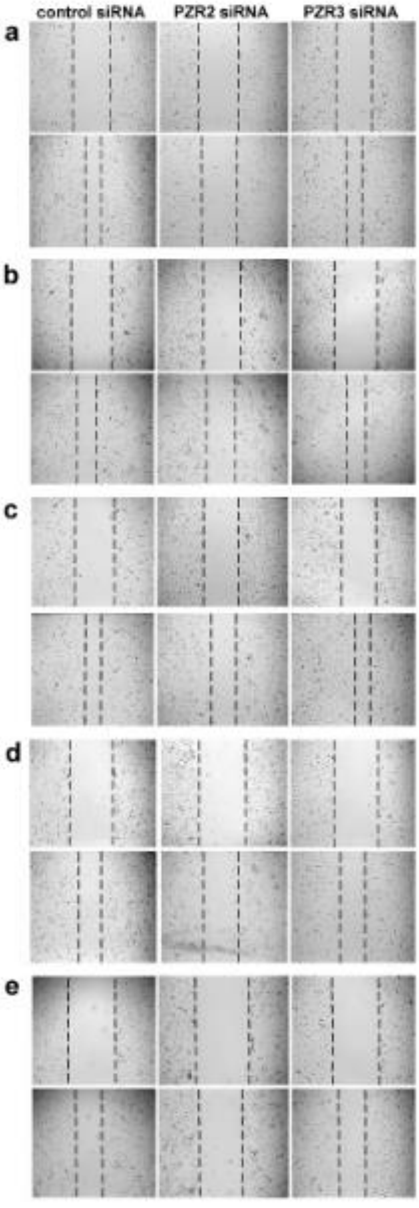

Supplementary Figure S4. siRNA knockdown of human PZR and PZRb for spreading and migration assays: Histograms show the relative knockdown of human PZR expression, after staining of hBM MSCs treated with siRNAs against *PZR* and *PZRb* or the control siRNAs with the WM78 mAb, followed by Alexa-488 goat anti-mIgG1 and prior to spreading (**A**) and migration (**D**) assays. These were compared to sham transfected cells, which were normalized to 100%. Values represent mean±S.E.M. (*n* = 3 independent experiments using 3 different batches of hBM MSCs). B and **E**) Effects of siRNA treatments on proliferation and viability of hBM MSCs isoforms prior to cell spreading (**B**) and migration (**E**) assays respectively. At day one, 2×10^5 hBM MSCs were transfected with the respective siRNAs and the cell count using a hemocytometer and cell viability were determined after 3 days. Values represent mean ± S.E.M. for three independent experiments using 3 different batches of hBM MSCs. Viability was defined as negative staining for the ToPro-3 dye. Phase contrast images of hBM MSCs treated with *PZR2*-, *PZR3*- or control siRNAs as assayed in the spreading (**C**) or migration (**F**) assays on (a) fibronectin, (b) vitronectin, (c) collagen I, (d) collagen IV and (e) laminin. For (**F**), migration was recorded at 0h (upper panel) and 20h (bottom panel) after initiating the migration assay.

Supplementary Figure S5. Roubelakis et al. 2020

A

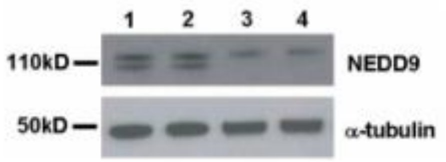

B

siRNA	siRNA specificity	Cell Count (x10^5) following siRNA Treatment (1)	% Cell viability following siRNA Treatment (2)
Nil	Nil	3.14±0.3	97.1±1.6
NEDD9-1	NEDD9	3.02±0.5	94.2±4.4
NEDD9-2	NEDD9	3.04±0.4	94.7±4.3
control siRNA	Nil	3.18±0.2	96.5±1.5

C

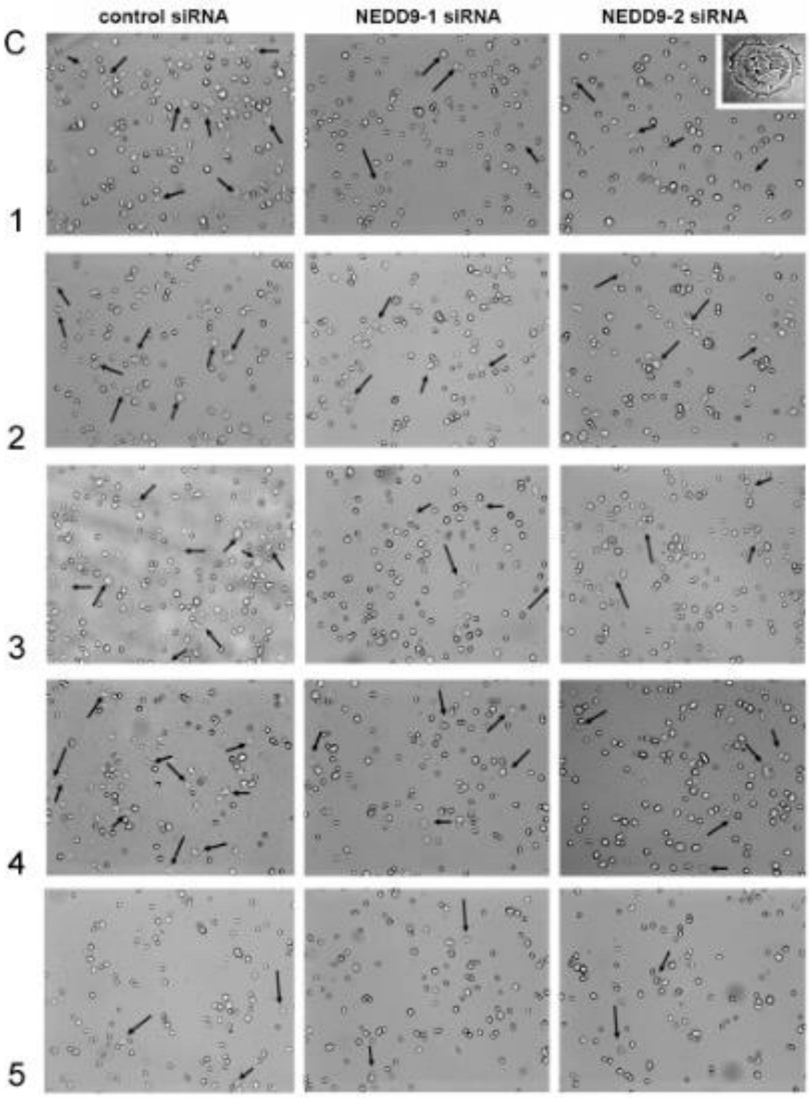

Supplementary Figure S5. siRNA knockdown of NEDD9 in hBM MSC prior to the cell spreading assay: **A**) Western blot for NEDD9 of hBM MSCs that were either sham transfected (lane 1) or transfected with control (lane 2) or *NEDD9-1* and *NEDD9-2* siRNAs (lanes 3 and 4), using the mouse anti-human NEDD9 mAb (2G9) and α-tubulin developed with HRP-goat anti-mouse IgG1 as a loading control (n = 3 independent experiments using 3 different batches of hBM MSCs). **B**) At day one, 2 × 10^5 hBM MSCs were transfected with the respective siRNAs and the cell count using a hemocytometer and cell viability was determined after 2 days. Values represent mean±S.E.M. for three independent experiments using 3 different batches of hBM MSCs. Viability was defined as negative staining for the ToPro-3 dye. **C**) NEDD9-dependent spreading of hBM MSCs on ECM. hBM MSCs treated with *NEDD9-1*, *NEDD9-2* or control siRNAs were allowed to adhere and spread on ECM molecules at 37 °C for 60min, at which time they were imaged using phase-contrast microscopy. NEDD9-dependent spreading of hBM MSCs on 1) FN, 2) VN, 3) COL-I, 4) COL-IV, and 5) LN. Arrows denote examples of cells that have spread. Inset shows a higher magnification of a cell that had spread on FN.

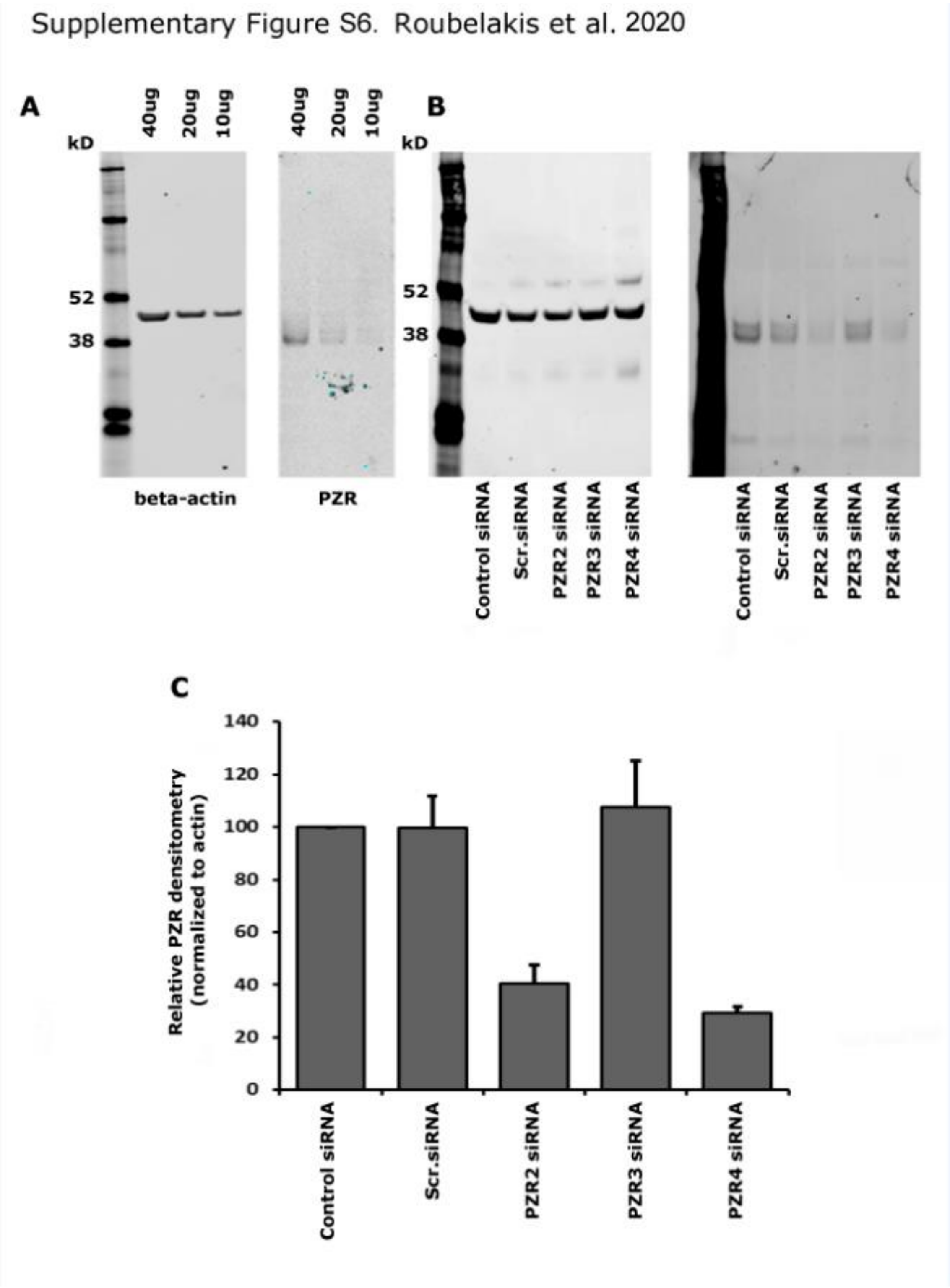

Supplementary Figure S6. Representative Western blots for PZR in hBM MSCs in the absence and presence of siRNAs: (**A**) hBM MSC lysates (10–40μg) analyzed for beta actin as the loading control or with rabbit anti-human PZR antibody. B) hBM MSCs were treated with control, pooled scrambled, *PZR2, PZR3* and *PZR4* siRNAs and Western blots of cell lysates analyzed with anti-beta actin (left) or rabbit anti-human PZR (right) and IRDye secondary antibodies. **C**) Densitometry plots of (**B**) comparing PZR to beta actin immunoblots and with the control siRNA blot normalized to 100% (n = 3 independent experiments using 3 different batches of hBM MSCs). Rainbow markers are shown for each set of blots. Statistical analysis was compared to the Scr.siRNAs and siRNA controls using one way ANOVA ($p < 0.001$) and Tukey's HSD post hoc test. PZR2 and PZR4 siRNA groups

were significantly different to the Scr.siRNA ($p < 0.001$; $p < 0.0005$ respectively) and Control siRNA groups ($p < 0.001$; $p < 0.0005$ respectively).
